# N, S Co‐Doped Carbon Quantum Dots‐Riboflavin Composite Photosensitizers for Enhanced Iontophoresis‐Assisted Corneal Cross‐Linking

**DOI:** 10.1002/advs.202510396

**Published:** 2026-05-05

**Authors:** Tinghong Xu, Yong Liu, Wenjing Zhang, Qiuruo Jiang, Yi Jin, Liangzheng Lin, Lumeng Wang, Zhanhao Gu, Mimi Lin, Shihao Chen

**Affiliations:** ^1^ School of Ophthalmology and Optometry School of Biomedical Engineering Wenzhou Medical University Wenzhou Zhejiang China; ^2^ Laboratory of Novel Optoelectronic Technology for Ophthalmic Devices (NOTOD) National Engineering Research Center of Ophthalmology and Optometry Eye Hospital Wenzhou Medical University Wenzhou Zhejiang China; ^3^ The Institute of Ocular Biomechanics National Clinical Research Center for Ocular Diseases Eye Hospital Wenzhou Medical University Wenzhou Zhejiang China

**Keywords:** carbon quantum dots, charge transfer, corneal cross‐linking, iontophoresis, NS‐CQDs‐RF composite photosensitizers, reactive oxygen species

## Abstract

Iontophoresis‐assisted corneal cross‐linking (I‐CXL) is an emerging transepithelial technique for treating keratoconus. I‐CXL employs a small electric current to enhance the corneal penetration of riboflavin 5′‐phosphate sodium (RF), avoiding the de‐epithelialization side effects associated with conventional CXL (C‐CXL). However, I‐CXL is less effective than C‐CXL due to insufficient RF surface charge hindering iontophoresis response and absorption efficiency, as well as inadequate reactive oxygen species (ROS) generation in the corneal stroma. This work reports the development of an innovative composite photosensitizer (NS‐CQDs‐RF), utilizing N, S co‐doped carbon quantum dots (NS‐CQDs) as nanocarriers, to overcome the bottlenecks facing I‐CXL. Experimental results show that the as‐synthesized NS‐CQDs‐RF composites exhibit a remarkable electric field response, enhanced RF corneal permeability, high ROS production efficiency, and excellent biocompatibility. Notably, I‐CXL incorporating NS‐CQDs‐RF significantly improves corneal biomechanical stability, outperforming C‐CXL while reducing total UVA irradiation energy without compromising efficacy. Density Functional Theory (DFT) calculations reveal that the optimized electronic structure and favorable energetics between NS‐CQDs and RF drive efficient charge transfer, amplifying ROS generation. These findings highlight the superiority of the NS‐CQDs‐RF composite photosensitizers in I‐CXL, offering a novel strategy for future keratoconus treatment.

## Graphical abstract

This study explores the use of N, S co‐doped carbon quantum dots (NS‐CQDs) as carriers for riboflavin, creating NS‐CQDs‐RF composite photosensitizers. These composites improve riboflavin absorption, enhance ROS generation efficiency, and preserve corneal epithelium integrity. The findings demonstrate superior treatment efficacy over conventional methods, offering a safer and more effective alternative for keratoconus therapy.

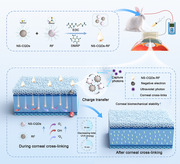

## 1 Introduction

Keratoconus (KC) is a common ectatic corneal disorder characterized by its bilateral, asymmetric, progressive, multifactorial, and non‐inflammatory features, which can lead to severe vision impairment or even legal blindness [1, 2]. It represents the leading global indication for corneal transplantation surgery, with an incidence of 1 in 2000 in the general population, and even higher among young adults [3]. Corneal collagen cross‐linking (CXL) is the primary treatment to stabilize KC progression [4]. CXL utilizes riboflavin 5′‐phosphate sodium (RF) as a photosensitizer; upon exposure to ultraviolet A (UVA) light at 370 nm, RF produces reactive oxygen species (ROS) within the corneal stroma via Type I and II photochemical reactions [5]. This process promotes covalent bonding between collagen molecules as well as between collagen molecules and proteoglycans, resulting in biomechanical and biochemical strengthening that ultimately halts the progression of KC [6, 7].

Epithelium‐off corneal collagen cross‐linking, also known as conventional CXL (C‐CXL), was first reported by Wollensak et al. in 2003 and is recognized as the gold standard for treating KC [8]. This procedure involves removing the corneal epithelium, instilling RF solution, and then applying UVA irradiation to induce cross‐linking [9]. This technique has demonstrated significant efficacy and long‐term stabilization of progressive KC [10]. However, complications associated with the epithelial debridement during C‐CXL have been reported, including impaired epithelial healing, infectious keratitis, corneal scarring, and, in rare cases, resultant vision loss [11, 12]. Consequently, transepithelial corneal collagen cross‐linking (TE‐CXL), which avoids epithelial debridement, has emerged as a potential alternative [13].

Transepithelial cross‐linking with iontophoresis (I‐CXL) is a novel, non‐invasive technique that delivers negatively charged RF across the corneal epithelium driven by a small electric current in a significantly shorter time [14, 15]. Clinical research has shown that I‐CXL can slow down KC progression in most patients, improving the topographic and visual parameters [16, 17, 18]. However, animal studies have revealed that the intrastromal RF concentration achieved with I‐CXL is lower than that of C‐CXL due to the barrier function of the intact epithelium [19, 20]. Moreover, parameters obtained from I‐CXL, such as stromal demarcation line depth, biomechanical properties, and resistance to corneal collagenase digestion, are inferior to those of C‐CXL [20, 21, 22]. Additionally, long‐term clinical trials have demonstrated that KC progresses more rapidly after I‐CXL than after C‐CXL [16, 23]. It is hypothesized that these outcomes result from the inadequate surface charge distribution of RF (which limits its response to the iontophoresis current) and insufficient amounts of ROS within the corneal stroma. Therefore, the incorporation of RF with nanomaterials that possess high charge carrier density and catalytic ROS production capacity is needed.

Carbon quantum dots (CQDs) are photosensitizing nanoparticles predominantly composed of sp^2^/sp^3^ carbon networks, with sizes less than 10 nm [24, 25]. Since their initial report in 2004, CQDs have attracted intense attention for multiple applications in optoelectronics, catalysis, sensors, and biomedicine [26−28]. However, their potential in corneal cross‐linking has not yet been explored. Given their excellent physical, chemical, and biological properties [29], CQDs are promising candidates for CXL procedures. CQDs exhibit unique electron transfer properties resulting from their strong quantum confinement and edge effects [30−32], which facilitate electron transfer within the NS‐CQDs‐RF system, thereby increasing the negative surface charge of RF. Additionally, photoexcited CQDs have been found to generate singlet oxygen (^1^O_2_) via energy transfer and electron transfer pathways. Their interaction with surrounding molecular oxygen or H_2_O results in the generation of hydroxyl radical (·OH) and superoxide anion (·O_2_^−^) via electron–hole pairs and charge separation processes [33−35]. Thus, the incorporation of CQDs promotes the generation of additional ROS from NS‐CQDs‐RF in the corneal stroma under UVA irradiation. Moreover, the high specific surface area [36], honeycomb‐like sp^2^‐hybridized carbon framework [37], and active π–π stacking interactions of CQDs provide exceptional drug‐loading capability [38], high stability, and excellent biosafety. Furthermore, CQDs exhibit a broad absorption band covering the ultraviolet to visible wavelengths [39], specifically encompassing the 370 nm wavelength employed in the CXL procedure. Notably, doping CQDs with heteroatoms has been reported to significantly modulate their carrier density, chemical composition, and band gap, thereby enhancing their functional properties [40, 41]. Collectively, these unique properties render CQDs excellent nanocarriers for RF, thereby supporting their application in I‐CXL.

**FIGURE 1:**
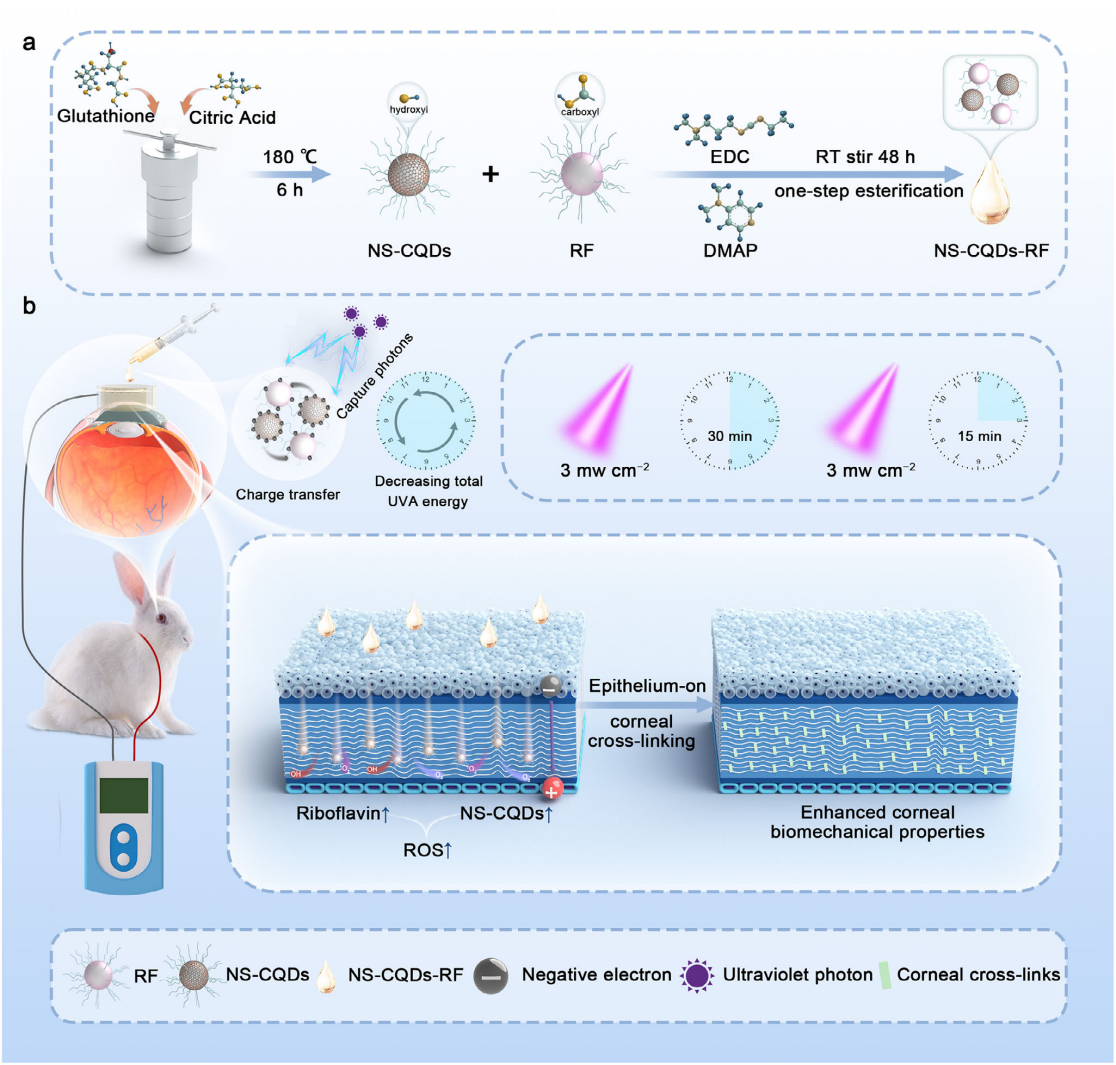
Schematic overview illustrating the preparation and application of NS‐CQDs‐RF composite photosensitizers for enhanced iontophoresis‐assisted corneal cross‐linking. (a) Schematic synthesis process of NS‐CQDs‐RF. (b) Diagrammatic illustration of the application procedure and mechanism of NS‐CQDs‐RF during iontophoresis‐assisted cross‐linking. EDC, 1‐ethyl‐3‐(3‐dimethylaminopropyl)carbodiimide; DMAP, 4‐dimethylaminopyridine; RT, room temperature; UVA, ultraviolet A; ROS, reactive oxygen species.

In this work, we present an innovative composite photosensitizer (NS‐CQDs‐RF) synthesized via a modified hydrothermal method, and subsequently fabricated through a one‐step esterification reaction (Figure 1a). The as‐synthesized NS‐CQDs‐RF exhibited enhanced electric field responsiveness, superior RF corneal permeability, higher ROS generation efficiency, and excellent biocompatibility. We then comprehensively evaluated the therapeutic efficacy of the NS‐CQDs‐RF composites as photosensitizers in I‐CXL. Our results demonstrated a higher stress–strain modulus, increased resistance to corneal collagenase digestion, and a deeper stromal demarcation line, indicative of an enhanced therapeutic effect. Furthermore, reducing the total UVA energy did not significantly alter the CXL effect, further highlighting the superiority of NS‐CQDs‐RF composites as photosensitizers for corneal cross‐linking applications (Figure 1b).

## 2 Results

### 2.1 Synthesis and Characterization of NS‐CQDs and NS‐CQDs‐RF Composite Photosensitizers

**FIGURE 2:**
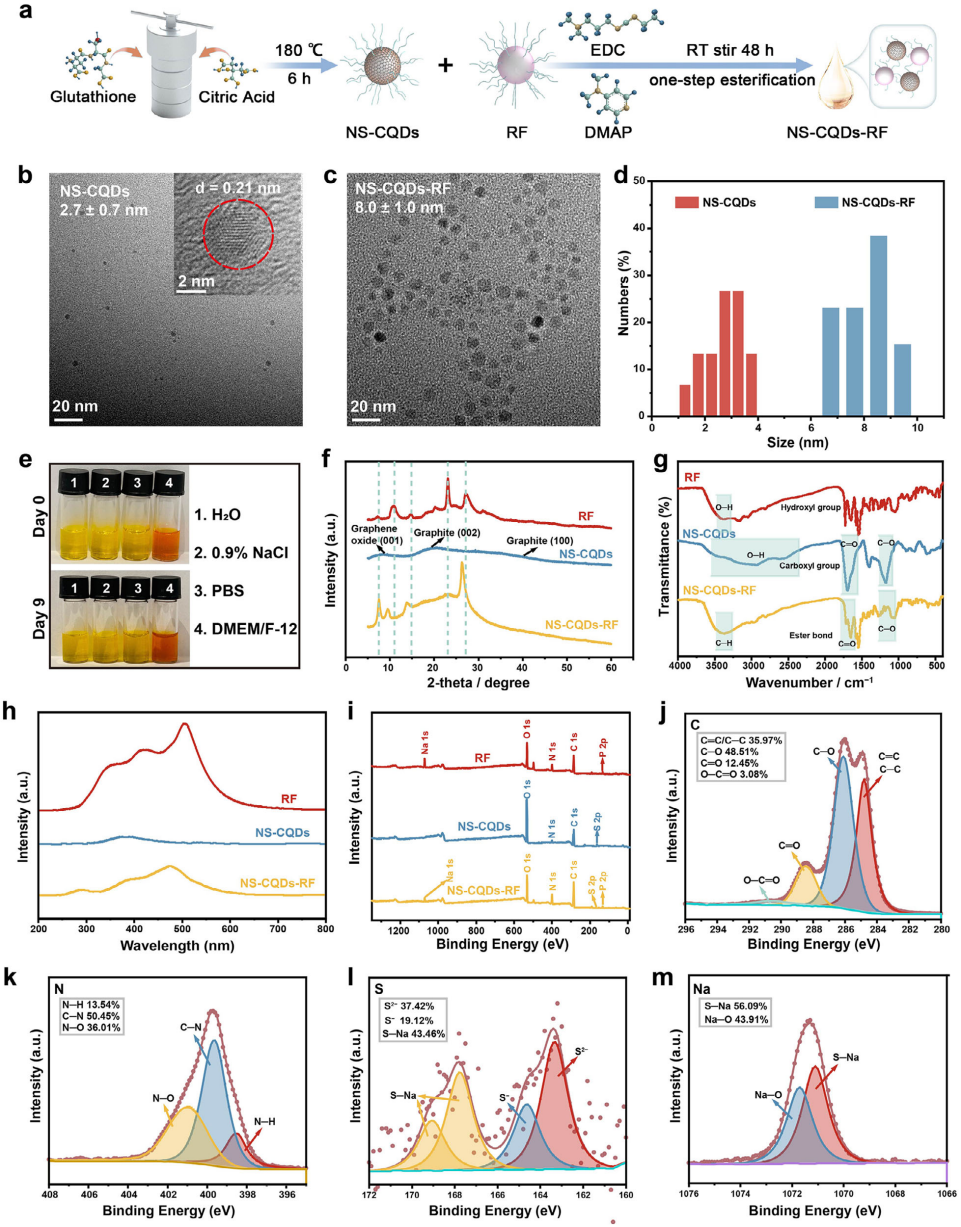
Synthesis and characterization of NS‐CQDs and NS‐CQDs‐RF composite photosensitizers. (a) An illustrative diagram of the processes involved in synthesizing NS‐CQDs and NS‐CQDs‐RF. (b) TEM image of NS‐CQDs; inset: HR‐TEM image of NS‐CQDs. (c) TEM image of NS‐CQDs‐RF. (d) Particle size distribution of NS‐CQDs and NS‐CQDs‐RF. (e) Digital photographs of NS‐CQDs‐RF in different solutions. (f) XRD patterns, (g) FT‐IR, (h) excitation spectra at 525 nm, and (i) XPS survey spectra of RF, NS‐CQDs, and NS‐CQDs‐RF. (j) High‐resolution XPS of C 1s, (k) high‐resolution XPS of N 1s, (l) high‐resolution XPS of S 2p, and (m) high‐resolution XPS of Na 1s for NS‐CQDs‐RF.

NS‐CQDs were synthesized via a simple hydrothermal method, employing citric acid as the carbon source and glutathione as the nitrogen and sulfur source (Figure 2a). The transmission electron microscopy (TEM) image of NS‐CQDs confirmed that the sample consisted of small‐sized, monodisperse nanoparticles, with a mean particle size of 2.7 ± 0.7 nm (Figure 2b,d). The high‐resolution TEM (HRTEM) image (inserted figure in Figure 2b) showed clear lattice fringes with a d‐spacing of 0.21 nm, corresponding to the (100) facet of graphite. The NS‐CQDs‐RF composite photosensitizers were synthesized through a one‐step esterification reaction (Figure 2a), with the RF encapsulation efficiency and loading efficiency recorded at approximately 76.7% and 79.1%, respectively (Figure S1). The TEM image demonstrated that the synthesized NS‐CQDs‐RF exhibited an increased particle size, with an average diameter of 8.0 ± 1.0 nm (Figure 2c,d). The zeta potential of NS‐CQDs‐RF in ddH_2_O stabilized at ≈−20 mV over 9 days, confirming the sufficient stability of the material (Figure S2). As displayed in Figure 2e, after 9 days, the NS‐CQDs‐RF composites maintained excellent stability in various solutions, including H_2_O, 0.9% sodium chloride (NaCl), phosphate‐buffered saline (PBS), and DMEM/F‐12 medium, further indicating prolonged resistance to aggregation and facilitating storage for subsequent cellular and animal experiments.

Subsequently, x‐ray diffraction patterns (XRD) of NS‐CQDs revealed characteristic peaks at 20° and 40°, assigned to the (002) and (100) facets of graphite, respectively, and a characteristic peak at 10.6°, corresponding to the (001) facet of graphene oxide. These findings confirm the successful synthesis of carbon quantum dots. After loading with RF, the XRD patterns of the NS‐CQDs‐RF composites displayed new peaks that, compared to RF alone, exhibited shifts in position and changes in intensity (Figure 2f). Furthermore, UV–vis absorption spectra were recorded for RF, NS‐CQDs, and the synthesized NS‐CQDs‐RF. As illustrated in Figure S3, the absorption peak of NS‐CQDs‐RF exhibited a notable blue shift compared to NS‐CQDs, indicating the formation of new chemical bonds within the NS‐CQDs‐RF composite system. Moreover, Fourier transform infrared (FTIR) analysis was carried out to characterize the products. Compared with NS‐CQDs, NS‐CQDs‐RF showed an increase in the absorption of C─H at 3375 cm^−1^ and a significant decrease in the absorption of C═O at 1716 cm^−1^ and C─O at 1180 cm^−1^, suggesting the hydroxyl group of RF and the carboxyl group of NS‐CQDs reacted to form an ester bond (Figure 2g).

Since both RF and NS‐CQDs possess excellent fluorescence properties and can produce reactive oxygen species under UVA irradiation, the excitation and emission spectra of RF (100 µg mL^−1^), NS‐CQDs, and NS‐CQDs‐RF (100 µg mL^−1^ RF) composites in double‐distilled water (ddH_2_O) were measured. As presented in Figure 2h, when monitored at 525 nm, RF displayed a broad ultraviolet excitation band ranging from 301 to 594 nm. Upon loading with NS‐CQDs, the form and range of the excitation spectral curve of NS‐CQDs‐RF composites were altered. In NS‐CQDs, the excitation peak was detected at 375 nm when monitored at 450 nm (Figure S4a). Upon excitation at 360 nm (a wavelength close to CXL), strong emission peaks were observed at 566 nm for RF, 457 nm for NS‐CQDs, and 556 nm for NS‐CQDs‐RF composites (Figure S4b). Additionally, when excited at 360 nm and 440 nm, the emission peaks of NS‐CQDs‐RF composites were shifted compared to those of RF alone due to the incorporation of NS‐CQDs (Figure S4b,c). Also, as showcased in Figure S5, the fluorescence excitation spectrum of RF overlapped with the emission spectrum of NS‐CQDs, which is consistent with the theory of Förster resonance energy transfer (FRET) of electronic excitation energy between appropriate donor–acceptor pairs [42]. These findings demonstrate the mutual influence of the fluorescence properties of NS‐CQDs and RF within the NS‐CQDs‐RF composites, revealing the presence of energy transfer mechanisms that enhance the composites’ ability to capture ultraviolet photons, thereby facilitating excitation reactions.

x‐ray photoelectron spectroscopy (XPS) analysis was conducted to determine the chemical compositions and surface electronic states of different elements in RF, NS‐CQDs, and NS‐CQDs‐RF composites. The elemental composition was analyzed by XPS in Figure 2i and Figure S6. The synthesized NS‐CQDs‐RF contained the typical elements Na and P from RF, as well as the characteristic element S from NS‐CQDs. The C 1s high‐resolution XPS spectra of NS‐CQDs‐RF were well fitted with four peaks at 284.8, 286.1, 288.4, and 290.6 eV, corresponding to C═C/C─C, C─O, C═O, and COOH, respectively (Figure 2j). Meanwhile, the N 1s peak was fitted to three peaks at 398.5, 399.6, and 401.0 eV, attributed to N─H, C─N, and N─O, respectively (Figure 2k). In the detailed scan of S 2p spectra, besides the S_2_^−^ and S^−^ peaks at 163.3 eV and 164.6 eV, respectively, the peaks at 167.8 eV and 169.1 eV were assigned to the S─Na bond, indicating the newly formed coordination bond in NS‐CQDs‐RF (Figure 2l) [43, 44]. In the Na 1s spectra, the fitted peak at 1071.7 eV was assigned to the Na─O bond, and the peak at 1071.1 eV was attributed to the S─Na bond (Figure 2m). In addition, peaks at 531.0, 532.4, and 534.7 eV corresponding to Na─O, C═O, and C─O phases, respectively, were monitored in the O 1s band (Figure S7a). The P 2p band was fitted with two peaks at 133.1 and 134.0 eV, corresponding to Na─P/P 2p_3/2_ and P─O/P 2p_1/2_ phases, respectively (Figure S7b).

In addition, undoped carbon quantum dots (CQDs), N‐doped CQDs (N‐CQDs), and S‐doped CQDs (S‐CQDs) were synthesized for comparison via a hydrothermal method. As shown in Figure S8, UV–vis spectra revealed characteristic absorption peaks near 330 nm for both N‐CQDs and NS‐CQDs, suggesting that nitrogen incorporation generates new surface defect states. Excitation and emission spectra further confirmed the modulation of energy bands by heteroatom doping. Compared to single‐element‐doped CQDs, the NS‐CQDs exhibited observable spectral shifts (Figure S9). These distinct optical features demonstrate that N, S co‐doping effectively reshapes the electronic structure of the carbon quantum dots, providing the foundation for efficient charge separation and ROS generation. Furthermore, XPS was employed to elucidate the surface chemical compositions. The high‐resolution C 1s spectra exhibited peaks at 284.0, 285.0, 286.6, and 288.8 eV, assigned to sp^2^ C, sp^3^ C, C─O/C─N/C─S, and C═O bonds, respectively (Figure S10a–c). The O 1s spectra displayed two characteristic peaks at 532.4 and 533.7 eV, corresponding to C═O and C─O bonds (Figure S10d–f). Notably, the higher proportion of C═O groups in NS‐CQDs compared to N‐CQDs and S‐CQDs indicates increased carboxyl group formation and an enhanced degree of oxidation (Table S1) [45]. Additionally, the N 1s spectra of N‐CQDs and NS‐CQDs were resolved into peaks at 400.2 and 402.0 eV for pyrrolic‐N and graphitic‐N, respectively (Figure S11a,b), while the S 2p spectra of S‐CQDs and NS‐CQDs showed characteristic S 2p_3/2_ and S 2p_1/2_ peaks at 163.6 and 164.9 eV (Figure S11c,d).

### 2.2 Characterization for Electron Transfer and Reactive Oxygen Species Generation in NS‐CQDs‐RF Composite Photosensitizers

**FIGURE 3:**
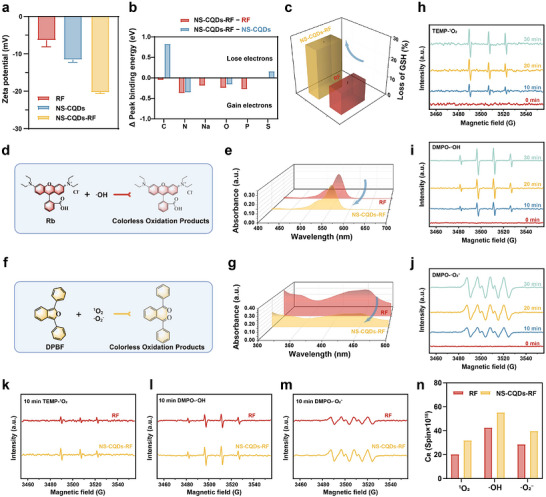
Increased surface negative charge and ROS generation efficiency of NS‐CQDs‐RF. (a) Zeta potential of RF, NS‐CQDs, and NS‐CQDs‐RF in ddH_2_O (pH 7, *n* = 3). (b) Peak binding energy differences of NS‐CQDs‐RF compared to RF and NS‐CQDs. (c) GSH consumptions of RF and NS‐CQDs‐RF under UVA irradiation (*n* = 6). (d) Schematic diagram of the oxidation reaction of Rb. (e) Rb consumptions of RF and NS‐CQDs‐RF under UVA irradiation. (f) Schematic diagram of the oxidation reaction of DPBF. (g) DPBF consumptions of RF and NS‐CQDs‐RF under UVA irradiation. (h–j) Electron paramagnetic resonance (EPR) spectra demonstrating ^1^O_2_, ·OH, and ·O_2_^−^ generation of NS‐CQDs‐RF under UVA irradiation. (k–m) EPR spectra of TEMP‐^1^O_2_, DMPO‐·OH, and DMPO‐·O_2_^−^ produced by free RF and NS‐CQDs‐RF in aqueous solutions after 10 min of UVA irradiation. (n) Concentrations of ^1^O_2_, ·OH, and ·O_2_^−^ (C_R_) at 10 min. Data are means ± SD. All experiments were conducted with three or more independent biological replicates per group. Diagrams of (d) and (f) were created with BioRender.com.

Zeta potential and x‐ray photoelectron spectroscopy (XPS) analysis were conducted to confirm the electron transfer from NS‐CQDs to RF in the NS‐CQDs‐RF composite system. As demonstrated in Figure 3a, the zeta potential of the synthesized NS‐CQDs‐RF composites was −20.1 mV, which was more negative than that of NS‐CQDs (−11.5 mV) and RF (−6.3 mV). The differences in peak binding energy (Δ peak binding energy) observed in the XPS spectra revealed that the peak binding energy of C 1s and S 2p in NS‐CQDs‐RF increased by 0.82 eV and 0.16 eV, respectively, compared to that in NS‐CQDs, indicating a tendency for NS‐CQDs to lose electrons. Conversely, compared to RF, the peak binding energy of Na 1s and P 2p in NS‐CQDs‐RF decreased by 0.19 eV and 0.27 eV, respectively, indicating that RF tended to gain electrons (Figure 3b). Furthermore, peaks at 167.8 eV and 169.1 eV, corresponding to the S─Na bond, were observed in the detailed scan of S 2p spectra, and a discernible peak at 1071.1 eV in the Na 1s spectra was also assigned to the S─Na bond (Figure 2l,m). These results demonstrate charge transfer from NS‐CQDs to RF, specifically the possibility of transfer from S elements in NS‐CQDs to Na elements in RF, which facilitates the response to the iontophoresis current in iontophoresis‐assisted CXL.

Numerous studies have demonstrated that during CXL procedures, the interaction between RF and ultraviolet irradiation induces ROS, resulting in the generation of additional covalent bonds between collagen molecules and enhancing corneal biomechanical properties [46, 47]. Moreover, photoexcited CQDs are also capable of generating ROS [33−35], suggesting that they may similarly increase the rigidity of the cornea by inducing covalent cross‐links between collagen fibrils. Therefore, investigating the ROS generation potential of NS‐CQDs‐RF compared to RF alone is critical for advancing subsequent in vivo animal studies. Initially, the GSH consumption capacity was investigated. As depicted in Figure 3c, under UVA irradiation, the GSH consumption of NS‐CQDs‐RF significantly increased in comparison to RF, indicating that NS‐CQDs enhance the oxidative stress level of the NS‐CQDs‐RF composites under these conditions. Further, the chemical probe Rhodamine B (Rb) was used to validate the yield of hydroxyl radical (·OH) (Figure 3d). As illustrated in Figure 3e, when exposed to UVA irradiation, the NS‐CQDs‐RF group exhibited significantly lower absorbance intensity than the RF group, indicating an enhanced ability to produce ·OH. Finally, 1,3‐diphenylisobenzofuran (DPBF) was employed as a trapping agent for singlet oxygen (^1^O_2_) and superoxide anion (·O_2_^−^) (Figure 3f). Figure 3g demonstrates that the NS‐CQDs‐RF group exhibits a more pronounced reduction in absorbance compared to the RF group upon UVA irradiation due to increased production of ^1^O_2_ and ·O_2_^−^.

In evaluating photoinduced ROS generation under UVA irradiation, the NS‐CQDs induced the highest GSH depletion compared to the other control groups (CQDs, N‐CQDs, S‐CQDs), indicating maximal oxidative stress (Figure S12a). Furthermore, when Rb and DPBF were used as probes, the NS‐CQDs induced the most pronounced absorbance decrease, demonstrating superior generation of ·OH, ^1^O_2_, and ·O_2_^−^ (Figure S12b,c). These results substantiate the necessity of N, S co‐doping for promoting charge transfer and enhanced ROS efficiency.

Importantly, electron paramagnetic resonance (EPR) experiments were performed to assess the production of ROS by NS‐CQDs‐RF solutions under UVA irradiation. The EPR signals were collected using 5,5‐dimethyl‐1‐pyrroline N‐oxide (DMPO) along with 2,2,6,6‐tetramethylpiperidine‐1‐oxyl (TEMP) as spin‐trapping agents to determine the types of ROS produced. As shown in Figure 3h–j, peaks corresponding to TEMP‐^1^O_2_, DMPO‐OH, and DMPO‐O_2_^−^ were identified, and the peak heights of each ROS increased with extended irradiation duration. To further compare the ROS generation capacities, EPR experiments were conducted using NS‐CQDs‐RF and free RF (both containing 100 µg mL^−1^ RF) at 10, 20, and 30 min post‐UVA irradiation. Notably, the signal intensities of the TEMP‐^1^O_2_, DMPO‐·OH, and DMPO‐·O_2_^−^ peaks generated by NS‐CQDs‐RF were consistently higher than those for free RF across all time points (Figure 3k–m and Figure S13a–c,e–g). Subsequent semi‐quantitative analysis revealed that the total spin concentrations of ^1^O_2_, ·OH, and ·O_2_^−^ produced by the NS‐CQDs‐RF system significantly exceeded those for free RF (Figure 3n and Figure S13d,h). Collectively, these findings demonstrate that the incorporation of RF with NS‐CQDs substantially enhances ROS generation.

### 2.3 Cytocompatibility and Permeating Capacity of NS‐CQDs‐RF In Vitro

**FIGURE 4:**
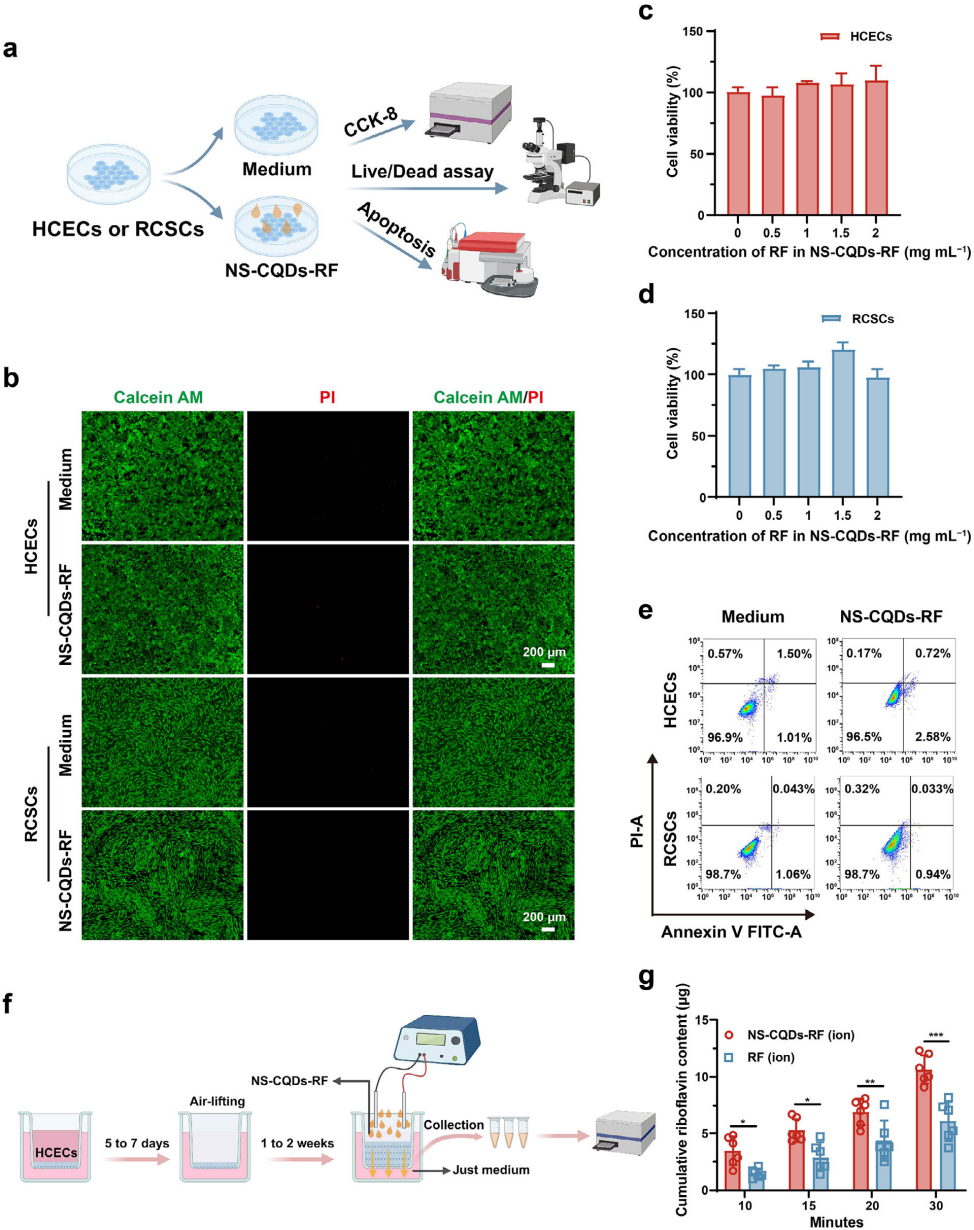
In vitro cytocompatibility and RF permeating capacity of NS‐CQDs‐RF. (a) Schematic diagram of experimental procedures to evaluate biocompatibility in HCEC and RCSC cells. (b) Fluorescent images of Live/dead staining on HCECs and RCSCs after incubation with NS‐CQDs‐RF for 24 h. (c) Cell viability of HCECs assessed by CCK‐8 kit after treatment with NS‐CQDs‐RF at different concentrations (*n* = 4). (d) Cell viability of RCSCs detected by CCK‐8 kit after treatment with NS‐CQDs‐RF at different concentrations (*n* = 4). (e) Representative flow cytometry scatter plots of HCECs and RCSCs treated with NS‐CQDs‐RF for 24 h with Annexin V/PI staining (*n* = 3). (f) Experimental scheme for RF permeability evaluation based on Transwell system. (g) Quantification of cumulative RF content in the lower chamber (*n* = 6). Data are means ± SD. Each point in the scatter plots represented one individual. All experiments were conducted with three or more independent biological replicates per group. ^*^ *p* < 0.05, ^**^ *p* < 0.01, and ^***^ *p* < 0.001. Statistical analysis was performed using two‐way ANOVA followed by Bonferroni's multiple comparisons test. ion, iontophoresis. Schematic diagrams of (a) and (f) were created with BioRender.com.

Live/dead cell staining, Cell counting kit‐8 (CCK‐8), and Annexin V apoptosis assay were conducted to evaluate the in vitro cytocompatibility of NS‐CQDs‐RF (Figure 4a). Representative live/dead staining images demonstrated that after 24 h of co‐incubation of NS‐CQDs‐RF solution (1 mg mL^−1^ RF) with HCECs and RCSCs, the majority of cells exhibited green fluorescence, indicating live cells, while only a few exhibited red fluorescence, indicating dead cells, similar to the results observed in the control medium group (Figure 4b). Additionally, CCK‐8 assay results indicated that NS‐CQDs‐RF exhibited no significant cytotoxicity toward HCECs and RCSCs at various concentrations (0–2 mg mL^−1^ RF) after 24 h of co‐incubation. Cell viability exceeded 90% in both cell types, suggesting excellent biocompatibility of NS‐CQDs‐RF (Figure 4c,d). Moreover, the Annexin V apoptosis assay revealed that the percentage of live cells was greater than 95% after 24 h of co‐incubation with NS‐CQDs‐RF solution (1 mg mL^−1^ RF) in both HCECs and RCSCs, which was in agreement with the results of Live/dead cell staining and CCK‐8 (Figure 4e). The percentages of living, early apoptotic, late apoptotic, and necrosis cells were comparable to those in the control medium group (Figure S14).

To assess the cumulative permeation content of RF in NS‐CQDs‐RF under electrical stimulation, a protocol for constructing a model of multilayer corneal epithelial cells and then conducting permeability experiments using the Transwell system was performed (Figure 4f). The model was considered successful when the transepithelial resistance (TER) value stabilized after reaching its maximum on day 10 (Figure S15a). As depicted in Figure 4g and Figure S15b, the NS‐CQDs‐RF (ion) group exhibited a significant increase in cumulative riboflavin content in the well at specific time intervals compared to the RF (ion) group, as measured by a microplate reader. These results collectively demonstrate that loading RF onto NS‐CQDs enhances its penetration at the cellular level, thereby providing a reference for subsequent animal experiments.

### 2.4 Evaluation of Corneal RF Absorption and ROS Generation In Vivo

**FIGURE 5:**
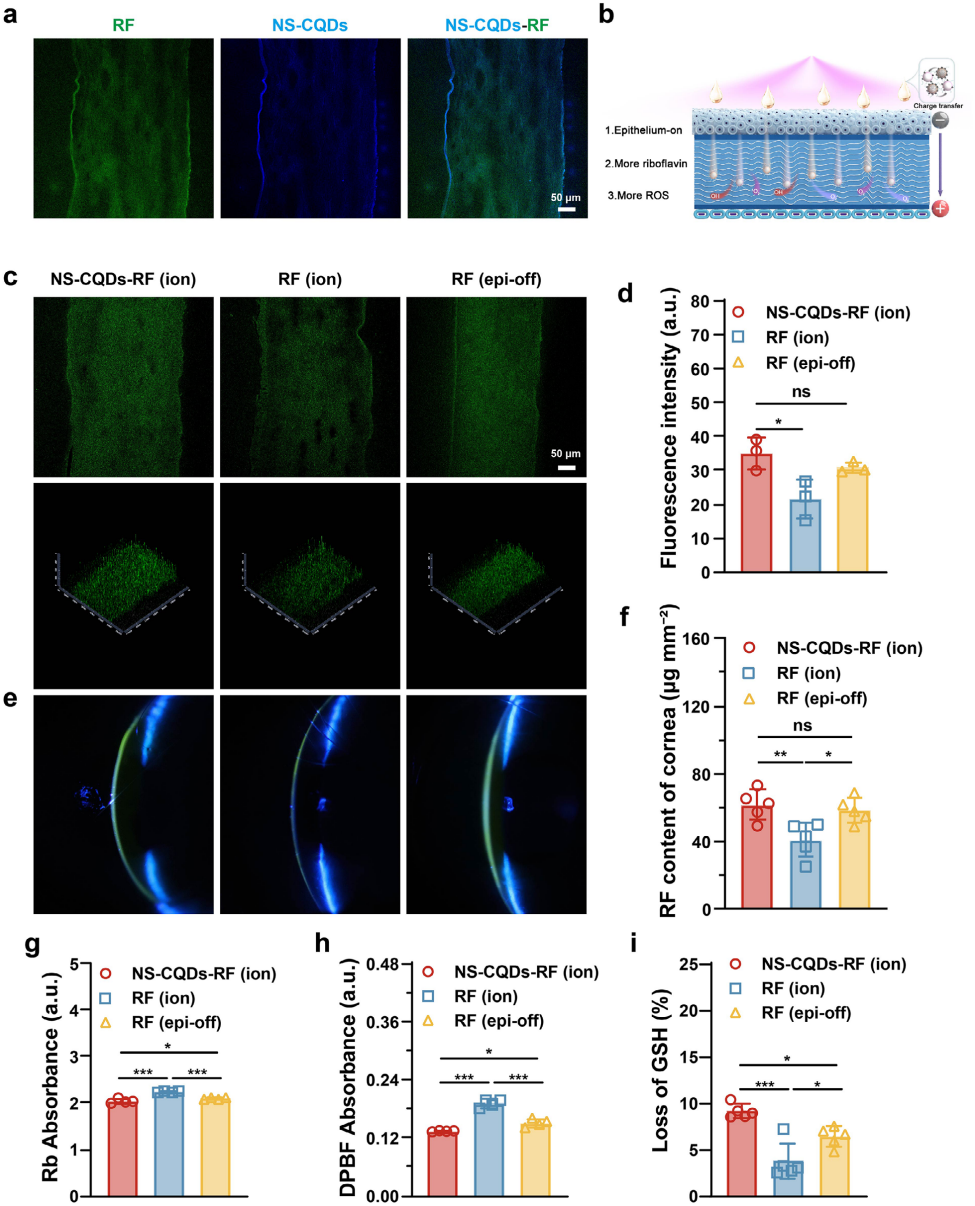
Enhanced corneal RF absorption and increased ROS generation in vivo. (a) Confocal fluorescence images in the NS‐CQDs‐RF (ion) group. The green fluorescence indicates the presence of RF, and the blue fluorescence indicates the presence of NS‐CQDs. (b) Schematic diagram of the multifunctional properties of NS‐CQDs‐RF for iontophoresis cross‐linking. (c,d) Confocal fluorescence images of corneal RF penetration and quantification of RF fluorescence in various groups (*n* = 3). (e) Cobalt blue slit lamp images of corneal RF penetration in different groups. (f) Quantification of RF content in central 9 mm corneal homogenates (*n* = 5). (g) Rb consumptions in different treatment groups (*n* = 4). (h) DPBF consumptions in various treatment groups (*n* = 4). (i) GSH consumption within different experimental treatments (*n* = 5). Data are means ± SD. Each point in the scatter plots represented one individual. All experiments were conducted with three or more independent biological replicates per group. ns, no significant difference; ^*^ *p* < 0.05, ^**^ *p* < 0.01, and ^***^ *p* < 0.001. Statistical analysis was performed using one‐way ANOVA followed by Tukey's multiple comparisons test. ion, iontophoresis; epi‐off, epithelium‐off.

To verify that NS‐CQDs and NS‐CQDs‐RF could infiltrate through the corneal epithelium into the corneal stroma via iontophoresis, frozen sections were observed by confocal microscopy. As shown in Figure 5a and Figure S16, blue fluorescence signals were observed in both the NS‐CQDs (ion) and NS‐CQDs‐RF (ion) groups, indicating that NS‐CQDs permeated the corneal epithelium by ion introduction. In addition, green fluorescence signals were detected, suggesting that RF penetrated the corneal epithelium utilizing NS‐CQDs as drug carriers (Figure 5a).

Having confirmed the high ROS generation efficiency and enhanced permeation capacity of NS‐CQDs‐RF in vitro, corneal RF absorption and ROS generation were subsequently evaluated in vivo (Figure 5b). To compare RF permeation in corneas under different treatments, confocal fluorescence microscopy, slit‐lamp observation under cobalt blue light, and measurements of RF content in corneal homogenates were performed, and the results were presented in Figure 5c–f.

Confocal fluorescence images of rabbit corneas presoaked with various samples were first evaluated. Compared to the RF (ion) group, the NS‐CQDs‐RF (ion) group displayed a significant increase in green fluorescence signal, reaching levels comparable to the RF (epi‐off) group (Figure 5c). Quantification for the fluorescent intensity results also confirmed the above observation (Figure 5d). Then, cobalt blue slit‐lamp photos of rabbit corneas after different treatments were captured. The NS‐CQDs‐RF (ion) group showed higher signal intensity than the RF (ion) group and was comparable to the RF (epi‐off) group (Figure 5e). Meanwhile, the RF content in corneal homogenates from different groups was determined by microplate reader assay. As shown in Figure 5f, the RF content in the NS‐CQDs‐RF (ion) group was approximately equivalent to that in the RF (epi‐off) group and considerably higher than that in the RF (ion) group. These results indicate that the synthesized NS‐CQDs‐RF composite photosensitizers exhibit enhanced permeability through the corneal epithelial barrier, achieving RF concentrations in the corneal stroma comparable to those observed in the RF (epi‐off) group.

The concentration of ROS in the stroma is significantly correlated with the CXL effect. To assess ROS levels, the central 9 mm of the corneal stroma was homogenized and exposed to 3 mW cm^−2^ UVA light for 30 min in the presence of ROS trapping agents Rb, DPBF, and GSH, and measurements were then taken using a microplate reader. The NS‐CQDs‐RF (ion) group exhibited the lowest Rb absorbance value, followed by the RF (epi‐off) group and finally the RF (ion) group (Figure 5g). The results indicate that the corneal hydroxyl radical concentration is highest in the NS‐CQDs‐RF (ion) group among the three groups tested. Meanwhile, Figure 5h demonstrates that the NS‐CQDs‐RF (ion) group exhibits the lowest DPBF absorbance value in comparison to the other two groups, indicating the highest production of singlet oxygen and superoxide anion. Finally, to evaluate the total ROS content in the corneal stroma, a GSH consumption experiment was performed. As illustrated in Figure 5i, the loss of GSH (%) in the cornea for the three groups was: NS‐CQDs‐RF (ion) > RF (epi‐off) > RF (ion). These results indicate that the synthesized NS‐CQDs‐RF composite photosensitizers possess enhanced ROS generation capability. Collectively, these findings establish a foundational basis for subsequent animal experiments aimed at evaluating the I‐CXL effect using NS‐CQDs‐RF composites as photosensitizers.

### 2.5 I‐CXL Efficacy Using NS‐CQDs‐RF Composite Photosensitizers In Vivo

**FIGURE 6:**
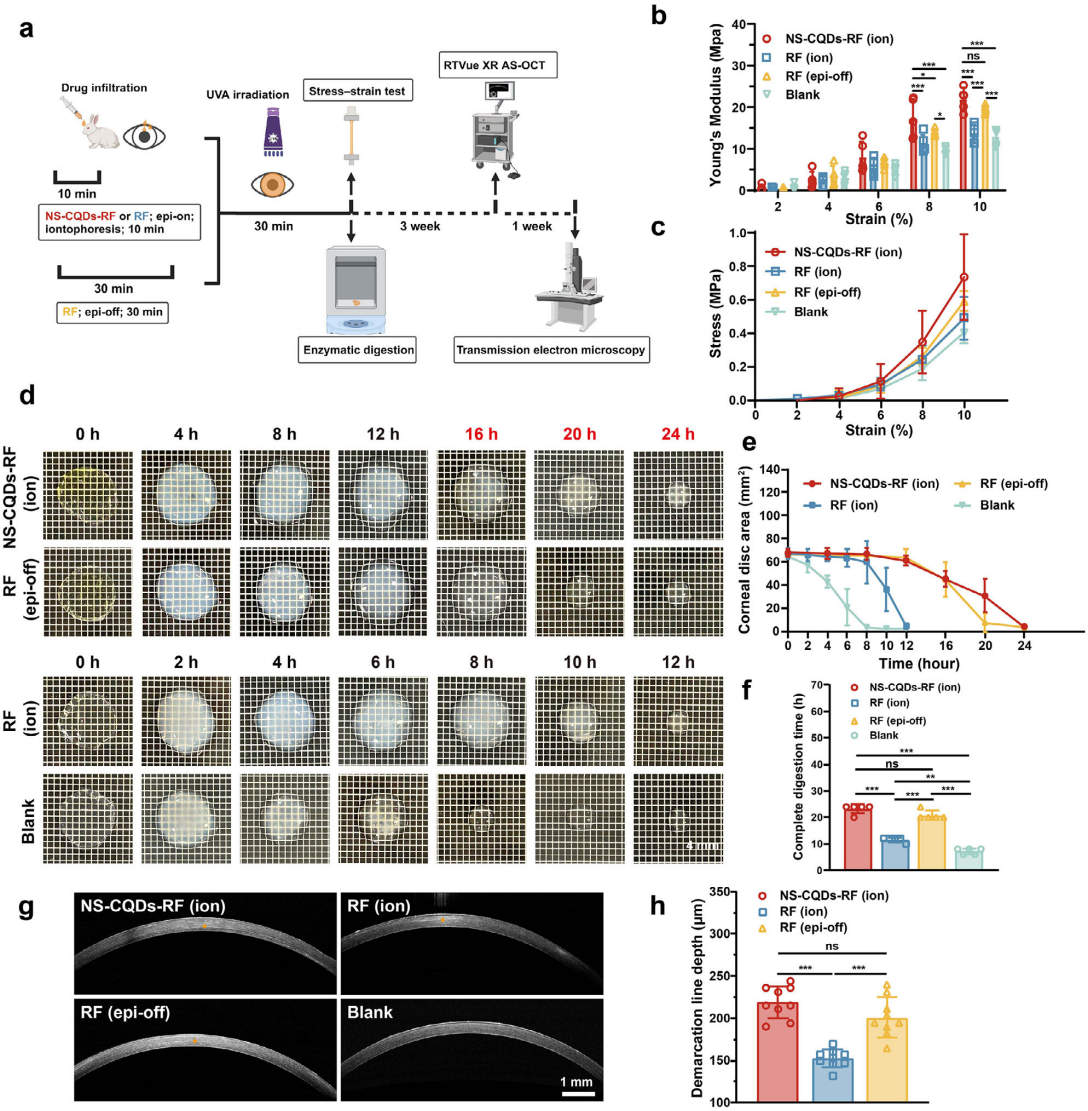
In vivo CXL evaluation. (a) Flow chart of CXL evaluation experiments. (b,c) Biomechanical properties of corneas with different treatments (*n* = 5). (d) Images of corneal tissue residues after undergoing enzymatic digestion at different times. (e) Graph of corneal disc area over time (*n* = 5). (f) Complete digestion time in different groups (*n* = 5). (g) Corneal stromal demarcation line visualized via high‐resolution AS‐OCT scanning 3 weeks after CXL. (h) The central corneal stromal demarcation line depth in different groups (*n* = 9). Data are means ± SD. Each point in scatter plots represented one individual. All experiments were conducted with three or more independent biological replicates per group. ns, no significant difference; ^*^ *p* < 0.05, ^**^ *p* < 0.01, and ^***^ *p* < 0.001. Statistical analysis was performed using two‐way ANOVA followed by Tukey's multiple comparisons test (b) and one‐way ANOVA followed by Tukey's multiple comparisons test (f, h). ion, iontophoresis; epi‐off, epithelium‐off. The schematic diagram of (a) was created with BioRender.com.

An illustration of the experimental procedure employed to evaluate the efficacy of CXL is presented in Figure 6a. Initially, to evaluate the biomechanical properties of the different groups, stress–strain measurements were performed on corneal strips. The NS‐CQDs‐RF (ion) group exhibited significantly greater stress and Young′s modulus at strains of 8% and 10% compared to the RF (ion) and Blank groups. Additionally, at these strain levels, the NS‐CQDs‐RF (ion) group showed slightly greater stress and Young′s modulus than the RF (epi‐off) group (Figure 6b,c). The above results indicate that applying NS‐CQDs‐RF composite photosensitizers to I‐CXL yields the greatest biomechanical enhancement compared to the other groups.

In the enzymatic digestion of collagen experiments, a 9 mm corneal disc was incubated with 2 mL of 0.1% Collagenase Type II solution at 200 rpm and 37°C for 24 h. Following digestion, photographs of the corneal disc residues were taken (Figure 6d–f). The residual corneal disc area curve showed that the NS‐CQDs‐RF (ion) group retained approximately 50% of its original size after 20 h of digestion, with complete digestion occurring at 24 h. By comparison, the RF (epi‐off) group was completely digested after 20 h, and the RF (ion) group was fully digested after 12 h (Figure 6d,e). As illustrated in Figure 6f, the complete digestion time for the four groups was: NS‐CQDs‐RF (ion) > RF (epi‐off) > RF (ion) > Blank. These results demonstrate that the application of NS‐CQDs‐RF composite photosensitizers prolongs the dissolution time compared to the other groups, indicating that loading NS‐CQDs enhances the efficacy of CXL.

For a comprehensive evaluation of I‐CXL using NS‐CQDs‐RF composites, the stromal demarcation line was visualized using anterior segment optical coherence tomography (AS‐OCT). Three weeks after CXL, AS‐OCT identified a densely compact, highly reflective linear region within the stroma. Figure 6g illustrates that the NS‐CQDs‐RF (ion) exhibits the deepest stromal demarcation line compared with the other groups. Additionally, the mean depth of the central stromal demarcation line was 219.1 ± 19.1 µm (SD) in the NS‐CQDs‐RF (ion) group, 152.2 ± 10.6 µm (SD) in the RF (ion) group, and 201.1 ± 23.5 µm (SD) in the RF (epi‐off) group (Figure 6h). Statistical analysis of the demarcation line depth (1 mm to the right and left of the center), as shown in Figure S17, also indicated similar results.

It has been reported that collagen fibril organization in the stroma is closely correlated with the biomechanical properties of the cornea [48]. Therefore, transmission electron microscopy was employed to determine collagen fibril diameter and interfibrillar spacing within the anterior stroma across different groups. As shown in Figure S18a, the collagen fiber density in the NS‐CQDs‐RF (ion) group was comparable to that in the RF (epi‐off) group and significantly higher than that in both the RF (ion) and Blank groups. The interfibrillar spacing and periphery‐to‐periphery spacing increased in the following order: NS‐CQDs‐RF (ion) group, RF (epi‐off) group, RF (ion) group, and Blank group (Figure S18b,c), suggesting that the NS‐CQDs‐RF (ion) group exhibits the most pronounced CXL efficacy. While collagen fibril diameter remained unchanged across all groups, this observation aligns with previous studies indicating that CXL treatment significantly reduces the interfibrillar spacing without notably affecting collagen fibril diameter (Figure S18d) [48, 49].

### 2.6 In Vivo Ocular and Systemic Biosafety of the NS‐CQDs‐RF Composite Photosensitizers

**FIGURE 7:**
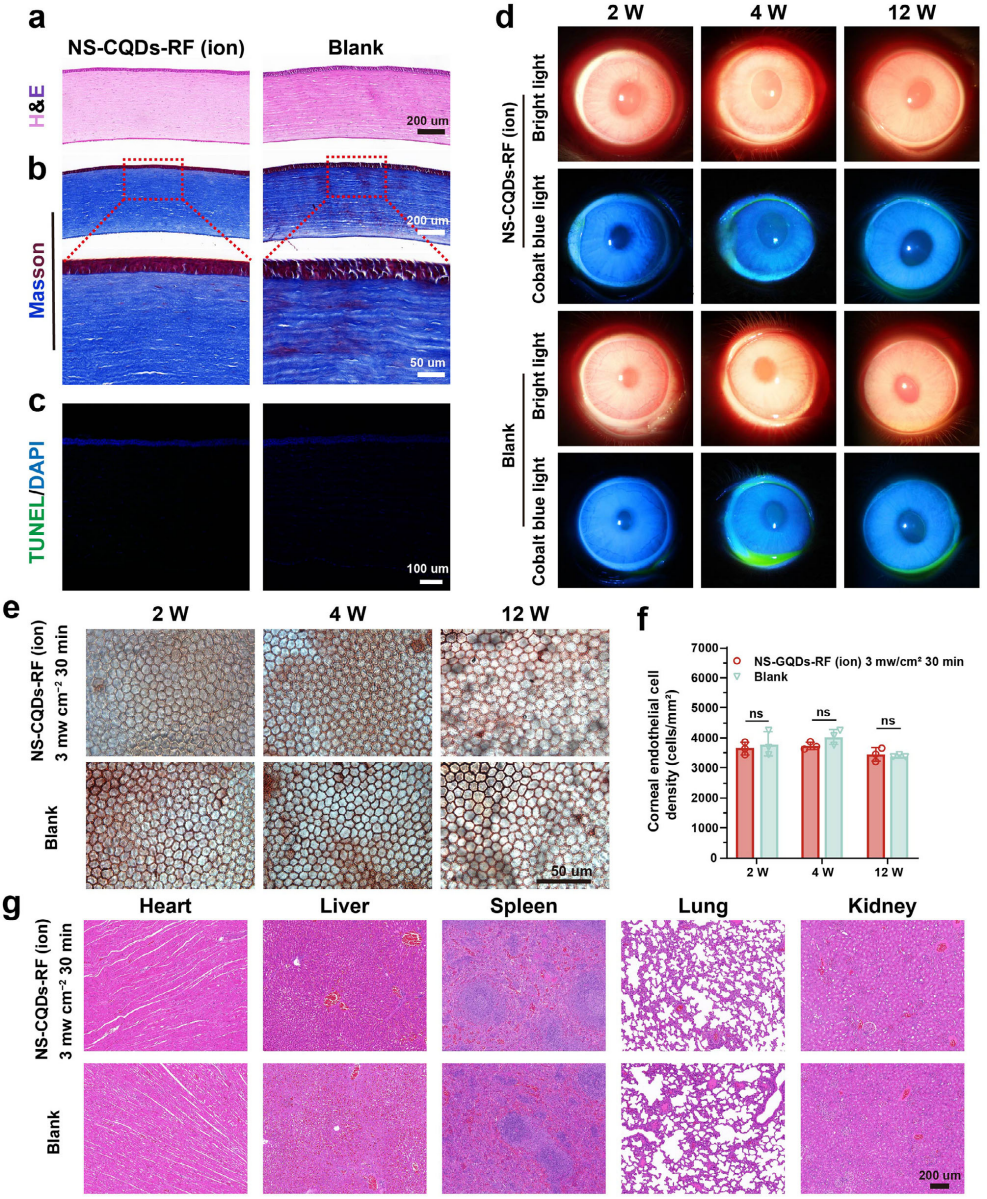
In vivo ocular and systemic biocompatibility evaluation. (a) Representative H&E staining images of corneas at 12 weeks post‐CXL treatment. (b) Representative Masson's trichrome staining images of corneas at 12 weeks post‐CXL treatment. (c) Representative TUNEL assay images of corneas at 12 weeks post‐CXL treatment. (d) Representative slit‐lamp biomicroscopy images of rabbit corneas taken at 2, 4, and 12 weeks post‐CXL. (e) Representative endothelium staining (ES) images in the NS‐CQDs‐RF (ion) and Blank groups at 2, 4, and 12 weeks post‐CXL. (f) Statistical results of corneal endothelial cell density in ES photos (*n* = 3). (g) Representative H&E staining images of major organs (heart, liver, spleen, lung, and kidney) harvested from the NS‐CQDs‐RF (ion) and Blank groups at 12 weeks post‐treatment. Data are means ± SD. Each point in the scatter plots represented one individual. All experiments were conducted with three or more independent biological replicates per group. ns, no significant difference. Statistical analysis was performed using two‐way ANOVA followed by Bonferroni's multiple comparisons test. ion, iontophoresis; epi‐off, epithelium‐off.

The biocompatibility of I‐CXL using NS‐CQDs‐RF composite photosensitizers was comprehensively evaluated. Initially, hematoxylin and eosin (H&E) staining at 2, 4, and 12 weeks post‐CXL showed that the corneal structure of the NS‐CQDs‐RF (ion) group remained as clear as that of the Blank group. The thickness of each corneal layer was not significantly altered; corneal epithelial and stromal cells were uniformly distributed without visible damage, and inflammatory cell counts showed no significant increase (Figure 7a and Figure S19). Similarly, Masson's trichrome staining at the same time points indicated that collagen fibers remained orderly with no structural damage (Figure 7b and Figure S20). To assess apoptotic DNA fragmentation, a TdT‐mediated dUTP nick end labeling (TUNEL) assay was conducted, and the results were depicted in Figure 7c and Figure S21. No TUNEL‐positive cells (green fluorescence) were identified in the NS‐CQDs‐RF (ion) group, demonstrating that apoptosis is not significantly induced, similar to the Blank group. Slit lamp biomicroscopy showed that the corneal epithelium was smooth, and no visible corneal punctate staining spots (green fluorescent spots) were observed in the NS‐CQDs‐RF (ion) group at 2, 4, and 12 weeks after CXL (Figure 7d). Furthermore, central corneal thickness, limbal thickness, and intraocular pressure exhibited no significant changes during the 4‐week monitoring period (Figure S22). In addition, to examine the effects on corneal endothelial cells, the endothelium was stained with Alizarin red S and trypan blue. As shown in Figure 7e, similar to the Blank group, endothelial cells in the NS‐CQDs‐RF (ion) group irradiated at 3 mW cm^−2^ for 30 min maintained their regular hexagonal morphology, displayed orderly alignment, and exhibited no blue staining, indicating that all cells preserve their physiologically healthy state. Statistical analysis of endothelium staining (ES) photos demonstrated that no significant difference was observed between the two groups in terms of endothelial cell density (Figure 7f). Finally, the systemic biosafety of the NS‐CQDs‐RF (ion) treatment was evaluated by harvesting major organs (heart, liver, spleen, lung, and kidney) at 12 weeks post‐treatment for H&E staining. As shown in Figure 7g, these organs in the treated group demonstrated no distinguishable changes compared with the Blank group. All of the above results indicate the exceptional in vivo biosafety of the NS‐CQDs‐RF composite photosensitizers, thereby laying a foundation for their clinical application.

### 2.7 I‐CXL Efficacy of Reduced Total UVA Energy Regimens

The above experiments indicated that the NS‐CQDs‐RF composite photosensitizers exhibited outstanding corneal RF penetration, enhanced ROS generation, and effective CXL outcomes. Therefore, to further evaluate the efficacy of NS‐CQDs‐RF in the I‐CXL procedure, a protocol reducing the total energy of UVA irradiation was tried.

**FIGURE 8:**
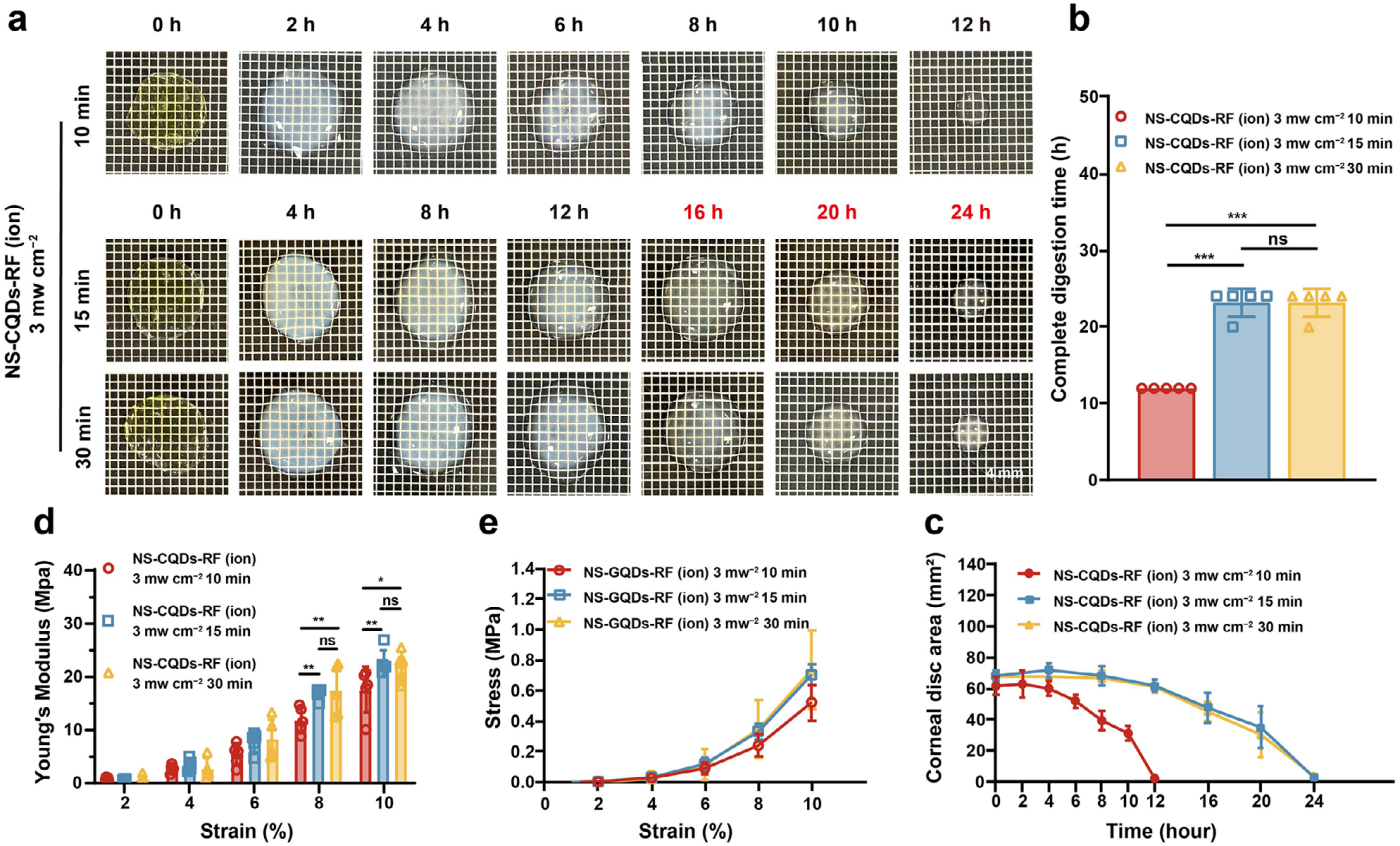
Efficacy evaluation of I‐CXL using NS‐CQDs‐RF composite photosensitizers under reduced UVA total energy protocols. (a) Images of corneal tissue residues after undergoing enzymatic digestion at different time points. (b) Complete digestion time in various groups (*n* = 5). (c) Comparison of corneal disc area over time among different groups (*n* = 5). (d,e) Comparison of biomechanical properties after different treatments (*n* = 5). Data are means ± SD. Each point in scatter plots represented one individual. All experiments were conducted with three or more independent biological replicates per group. ns, no significant difference; ^*^ *p* < 0.05, ^**^ *p* < 0.01, and ^***^ *p* < 0.001. Statistical analysis was performed using one‐way ANOVA followed by Tukey's multiple comparisons test (b) and two‐way ANOVA followed by Tukey's multiple comparisons test (d). ion, iontophoresis.

An experimental process, shown in Figure S23, is conducted in an attempt to reduce the duration of irradiation. After being presoaked with NS‐CQDs‐RF via iontophoresis for 10 min, the corneas were illuminated with solid‐state UVA sources for 10, 15, and 30 min, respectively, and then retrieved for further analysis. Representative images of the corneal tissue residues during enzymatic digestion in the three groups were depicted in Figure 8a. In the NS‐CQDs‐RF (ion) 3 mW cm^−2^ 10 min group, it took approximately 12 h to almost completely dissolve. In comparison, the enzymatic digestion rates in the NS‐CQDs‐RF (ion) groups exposed to 3 mW cm^−2^ for 15 and 30 min were slower, requiring approximately 24 h for complete dissolution. Statistical analyses of the complete digestion time and corneal disc area, as shown in Figure 8b,c, also revealed similar findings. The stress–strain measurements were shown in Figure 8d,e. The stress and Young′s modulus of the NS‐CQDs‐RF (ion) 3 mW cm^−2^ 15 min group were nearly the same as those of the 30 min group, and both were higher than those of the 10 min group at strains of 8% and 10%. The above results indicate that reducing UVA irradiation time to 15 min produces the same biomechanical effect as 30 min, thus demonstrating the feasibility of reducing the total UVA energy when employing NS‐CQDs‐RF composites as photosensitizers for I‐CXL applications.

### 2.8 Density Functional Theory (DFT) Analysis of Charge Transfer and ^1^O_2_ Generation

**FIGURE 9:**
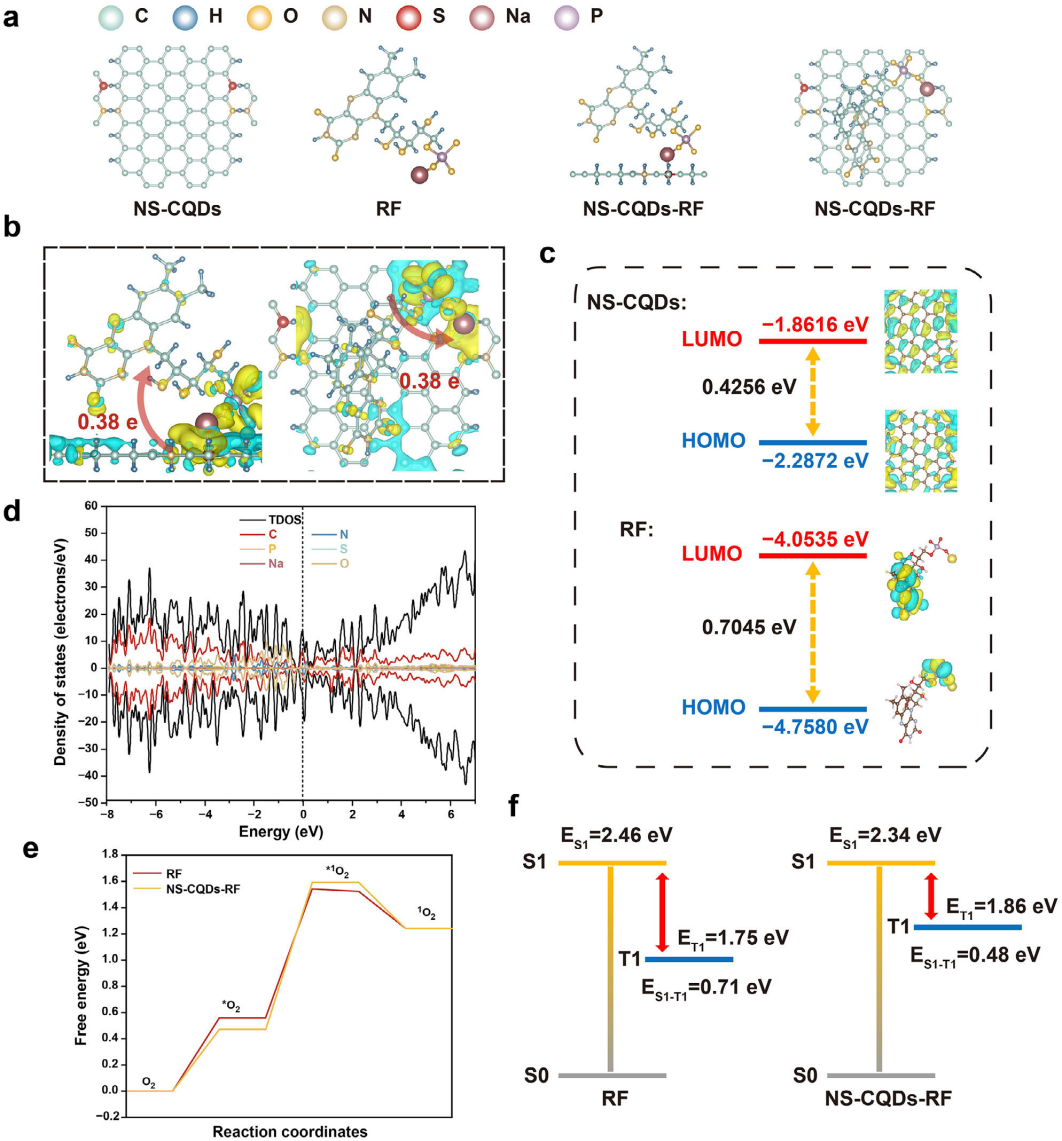
Density functional theory (DFT) insights into charge transfer and ^1^O_2_ generation in NS‐CQDs‐RF composites. (a) Molecular structure models of NS‐CQDs, RF, and the NS‐CQDs‐RF composites. (b) Differential charge density distribution analysis of NS‐CQDs‐RF. Yellow and cyan regions indicate charge accumulation and depletion, respectively. (c) HOMO and LUMO energy levels of NS‐CQDs and RF. (d) Total and partial density of states (TDOS and PDOS) of the NS‐CQDs‐RF composites. (e) Free‐energy diagrams for RF and the NS‐CQDs‐RF composites. (f) S1 and T1 energy levels of RF and the NS‐CQDs‐RF composites.

To elucidate the charge transfer from NS‐CQDs to RF and the underlying mechanism of ^1^O_2_ generation within the NS‐CQDs‐RF composite photosensitizers, molecular models for NS‐CQDs, RF, and the NS‐CQDs‐RF composites were constructed (Figure 9a). Differential charge density plots confirmed the existence of charge transfer between the NS‐CQDs and RF. Bader charge analysis revealed that the RF moiety partially gained electrons from the NS‐CQDs via S─Na bonds, with an estimated charge transfer of 0.38 eV (Figure 9b). As shown in Figure 9c, the favorable alignment between the higher HOMO level of NS‐CQDs (−2.2872 eV) and the lower LUMO level of RF (−4.0535 eV) provided a strong thermodynamic driving force for electron transfer from the donor (NS‐CQDs) to the acceptor (RF). Furthermore, total and partial density of states (TDOS/PDOS) analyses demonstrated that the composite interface possessed a substantial density of states near the Fermi level, ensuring continuous electronic states for efficient carrier separation (Figure 9d). These frontier states were primarily governed by the carbon skeleton and valence‐band O states, while heteroatoms (N, S, P) introduced localized band‐edge states to modulate charge distribution and further facilitate charge transfer.

Free energy profiles revealed that the NS‐CQDs‐RF composites possessed more favorable energetics for oxygen‐related reactions compared to pristine RF (Figure 9e). The lower O_2_ adsorption energy of NS‐CQDs‐RF (0.47 eV) facilitated the efficient capture and stabilization of ^*^O_2_ compared to that of RF (0.56 eV). Notably, the energy drop (0.35 eV) in NS‐CQDs‐RF during the ^*1^O_2_ to ^1^O_2_ desorption was significantly larger than that in RF (0.30 eV). This accelerated the release of surface‐bound singlet oxygen, thereby improving the yield of free ^1^O_2_. Furthermore, as illustrated in Figure 9f, the narrower singlet‐triplet energy gap (E_S1−T1_) of NS‐CQDs‐RF (0.48 eV), relative to that of RF (0.71 eV), enhanced spin‐orbit coupling (SOC) by facilitating spin mixing between the S1 and T1 states. Additionally, the higher T1 energy level of NS‐CQDs‐RF (1.86 eV), compared to that of RF (1.75 eV), provided a stronger thermodynamic driving force for energy transfer to O_2_. Ultimately, this optimal energy alignment and robust spin coupling synergistically maximize ^1^O_2_ generation.

## 3 Discussion

Keratoconus is a progressive ocular disorder characterized by corneal thinning and ectasia, severely compromising patients’ visual function and quality of life. Corneal collagen cross‐linking (CXL) is currently the primary clinical intervention to halt disease progression. However, conventional CXL (C‐CXL) carries inherent risks of postoperative pain, infection, and delayed wound healing. Meanwhile, conventional transepithelial CXL (TE‐CXL) suffers from limited efficacy due to poor RF permeation and insufficient ROS generation. Additionally, high‐dose ultraviolet irradiation poses potential cytotoxicity risks [50]. Recently, nanotechnology has emerged as a promising strategy to overcome these clinical limitations.

The primary obstacle to TE‐CXL is the corneal epithelial barrier, which restricts the penetration of the hydrophilic riboflavin 5′‐phosphate sodium [51]. Various nanocarrier systems have been developed to enhance RF permeation, including nanostructured lipid carriers (NLCs) [52], cyclodextrins [53], metal–organic frameworks (MOFs) [54], and magnetic nanoparticles (MNPs) [55]. Despite these innovations, their clinical translation remains hampered. Primarily, their large particle sizes and restricted transmembrane kinetics necessitate prolonged permeation times. Furthermore, most studies have focused solely on RF permeability, lacking systematic in vivo validation of biomechanical efficacy and long‐term biosafety. Concurrently, while emerging nanomaterial‐based photosensitizers, such as ruthenium‐based complexes [56], graphitic carbon nitride (g‐C_3_N_4_) [57], and lithium phenyl‐2,4,6‐trimethylbenzoylphosphinate (LAP) [58], have advanced CXL technology with high ROS yields, practical limitations restrict their clinical potential. Most platforms still rely on epithelial debridement and extended permeation periods, failing to achieve a truly noninvasive treatment. Finally, the underlying photophysical and photochemical synergistic mechanisms have not yet been fully elucidated, and comprehensive in vivo toxicological data remain limited.

To address these limitations, the NS‐CQDs‐RF system developed in this study achieves several technical breakthroughs. Utilizing the unique properties and mechanistic design of carbon quantum dots, this system demonstrates excellent transepithelial RF permeation, high ROS yields, and reliable long‐term in vivo biocompatibility. When combined with iontophoresis, this protocol completely preserves the corneal epithelium. Notably, it achieves a biomechanical strengthening effect superior to that of C‐CXL for the first time. Furthermore, it offers clear clinical advantages by requiring a shorter permeation time and a reduced total UVA dose.

Translating the outcomes of this work into clinical application requires careful consideration of anatomical differences. Unlike rabbit eyes, human corneas are thicker and feature a dense Bowman's layer, which restricts deep photosensitizer penetration [59]. However, the ultrasmall dimensions of the NS‐CQDs, combined with the driving force provided by iontophoresis, can effectively overcome this barrier. For future clinical application, we propose a safe and efficient framework focused on a transepithelial approach. NS‐CQDs‐RF would be applied via iontophoresis for 10 min, enabling effective drug delivery while avoiding the pain and infection risks associated with C‐CXL. Regarding the UVA dose, the high ROS yield of this system reduces the standard irradiation time from 30 to 15 min at 3 mW cm^−2^. This shorter exposure improves clinical efficiency, minimizes patient discomfort, and prevents toxicity to deep stromal and endothelial cells. Furthermore, in terms of treatment repeatability, preserving the epithelium and lowering total UVA energy ensures high safety, making repeated treatments feasible for progressive cases. Ultimately, large‐animal models and clinical trials are necessary to establish final guidelines [60]. Overall, this strategy achieves biomechanical strengthening comparable to that of the gold standard while keeping the epithelium intact, significantly advancing TE‐CXL technology.

Furthermore, this study highlights the value of theoretical calculations in understanding photophysical mechanisms. Future theoretical modeling will be a powerful tool for designing new ROS photosensitizers, enabling the computational screening and optimization of materials before chemical synthesis. Ultimately, this computation‐driven approach overcomes the limitations of traditional trial and error. It accelerates the development of safe and efficient next‐generation nanophotosensitizers, offering new therapeutic options for keratoconus and other ocular diseases.

In conclusion, this study presented the novel NS‐CQDs‐RF composite photosensitizers for application in transepithelial iontophoresis‐assisted cross‐linking to treat keratoconus. These composite photosensitizers efficiently responded to the iontophoresis electric field and enhanced riboflavin corneal absorption. By effectively capturing photons and facilitating electron transfer to generate reactive oxygen species, they improved the efficacy of TE‐CXL and mitigated ultraviolet‐induced damage. The innovative nanocomposite photosensitizers offer a therapeutic intervention for corneal ectasias, providing new insights for treatment in the field of keratoconus.

## 4 Experimental Section

### 4.1 Materials and Reagents

Citric acid, glutathione, urea, mercaptosuccinic acid (MSA), 1‐ethyl‐3‐(3‐dimethylaminopropyl)carbodiimide (EDC), 4‐dimethylaminopyridine (DMAP), Rhodamine B (Rb), 1,3‐diphenylisobenzofuran (DPBF), and Riboflavin 5′‐phosphate sodium (RF) were purchased from Aladdin (Shanghai, China). Collagenase Type II was purchased from Worthington Biochemical Corporation (Lakewood, NJ, USA). Cell Counting Kit‐8 was purchased from Dojindo (Japan). Calcein‐AM/PI double staining kit, Masson's trichrome stain kit, Alizarin red S, and Trypan blue were purchased from Solarbio (China). An Annexin V‐FITC/PI apoptosis detection kit was purchased from BD Biosciences. A one‐step TUNEL Assay Apoptosis Detection Kit and a TUNEL‐positive control kit were purchased from Beyotime (China).

### 4.2 Ethical Approval

All animal experiments were conducted in accordance with institutional guidelines and were approved by the Laboratory Animal Ethics Committee of Eye Hospital of Wenzhou Medical University (Approval Number: YSG23103002).

### 4.3 Synthesis of CQDs and Their Heteroatom‐Doped Variants

Following a modified method based on literature reports, NS‐CQDs were prepared [61, 62]. Typically, 5 g of citric acid and 1.5 g of glutathione were dissolved in 30 mL of double‐distilled water (ddH_2_O) with ultrasonic treatment. The resulting transparent substance was then transferred into a 100 mL stainless steel autoclave lined with Teflon, sealed, and heated at 180°C for 6 h in an electric oven, increasing the temperature at a rate of 10°C per minute. After the reaction, the autoclave was allowed to cool naturally to ambient temperature. The solution obtained was purified by passing it through a 50 nm filter to remove larger particles, followed by dialysis using a molecular weight cut‐off (MWCO) of 1000 Da. Finally, a pure NS‐CQDs solution was obtained. For comparison, control CQDs, N‐CQDs, and S‐CQDs were prepared using the same procedure but with different precursors: 6.5 g of citric acid for CQDs, 5.0 g of citric acid with 1.5 g of urea for N‐CQDs, and 5.0 g of citric acid with 1.5 g of mercaptosuccinic acid (MSA) for S‐CQDs [63, 64].

### 4.4 Synthesis of NS‐CQDs‐RF Composite Photosensitizers

In a typical synthesis of NS‐CQDs‐RF composite photosensitizers, 1 mL of NS‐CQDs solution (10 mg mL^−1^), 40 mg of riboflavin 5′‐phosphate sodium (RF), 28 mg of EDC, and a catalytic amount of DMAP were dissolved in 10 mL of PBS (1 M, pH 5). The mixture was stirred at ambient temperature for 48 h. Afterwards, pure NS‐CQDs‐RF composites were obtained by dialysis using a molecular weight cut‐off (MWCO) of 1000 Da.

### 4.5 Characterizations

TEM and HRTEM images were acquired using a JEM‐2100F instrument (JEOL, Japan). The zeta potential was determined by dynamic light scattering using a Malvern Zetasizer Nano ZS90 (Malvern Instruments Limited, UK). XRD patterns were recorded with a Rigaku SmartLab diffractometer (Rigaku, Japan). Ultraviolet–visible (UV–vis) absorption spectra were recorded with a UV‐3210 PC UV–visible spectrophotometer (China). The surface functional groups of the samples were recorded via FT‐IR spectroscopy over a range of 400 to 4000 cm^−1^, utilizing a Fourier transform infrared spectrometer (Thermo Fisher Scientific) with the KBr disk method. XPS measurements were performed on a Thermo Fisher Scientific Nexsa G2 XPS System. The excitation and emission spectra were measured by an F‐320 fluorescence spectrophotometer (Gangdong Sci. & Tech).

### 4.6 Hydroxyl Radical (·OH) Generation

To confirm the generation of ·OH by the NS‐CQDs‐RF composites, Rb was used as a chemical probe. In a typical experiment, 500 µL of NS‐CQDs‐RF solution (100 µg mL^−1^ RF) was mixed with 200 µL of Rb solution (1 mg mL^−1^, pH 6.5) and then exposed to UVA irradiation (3 mW cm^−2^ power density) for 30 min. Optical density (OD) values at wavelengths ranging from 400 to 700 nm were measured using a UV‐3210 PC UV–visible spectrophotometer (China). To demonstrate the synergistic effect of NS‐CQDs in NS‐CQDs‐RF composites, ·OH generation of the RF solution (100 µg mL^−1^) was monitored as a control using the same method. Furthermore, to compare the intrinsic ·OH generation capacities, the carbon dot variants (CQDs, N‐CQDs, S‐CQDs, and NS‐CQDs) were evaluated using an identical protocol at the same concentration of 10 µg mL^−1^.

### 4.7 Singlet Oxygen (^1^O_2_) and Superoxide Anion (·O_2_^−^) Generation

To detect the generation of ^1^O_2_ and ·O_2_^−^ by the NS‐CQDs‐RF composites, DPBF was utilized as a trapping agent. A working solution was prepared by dissolving DPBF (30 µM) in 3 mL of ethanol. Subsequently, 200 µL of the prepared DPBF solution was mixed with 500 µL of NS‐CQDs‐RF solution (100 µg mL^−1^ RF) and then irradiated with a 3 mW cm^−2^ UVA light source for 30 min. The absorption spectra of the DPBF mixture were recorded using a UV‐3210 PC UV–visible spectrophotometer (China). To illustrate the synergistic effect of NS‐CQDs in the NS‐CQDs‐RF composites, the same procedure was used to monitor the ^1^O_2_ and ·O_2_^−^ production in RF solution (100 µg mL^−1^) as a control. For a direct comparison of the intrinsic ^1^O_2_ and ·O_2_^−^ generation efficiencies, the four carbon dot variants (CQDs, N‐CQDs, S‐CQDs, and NS‐CQDs) were assessed under standardized conditions at a fixed concentration of 10 µg mL^−1^.

### 4.8 GSH Consumption Experiment

GSH consumption of NS‐CQDs‐RF was investigated using a DTNB Detection Kit (Leagene). GSH (50 µM) was added to an NS‐CQDs‐RF solution (10 µg mL^−1^ RF) and exposed to UVA irradiation (3 mW cm^−2^ power density) for 30 min. By adding 100 µM DTNB to the mixtures, a yellow substance was generated. Absorbance at 410 nm was measured by a microplate reader (Spectramax M5, Molecular Devices, USA). A GSH solution without NS‐CQDs‐RF and UVA irradiation served as a negative control for comparison. Using the same method, the GSH consumption of an RF solution (10 µg mL^−1^) was monitored to show the collaborative impact of NS‐CQDs in the NS‐CQDs‐RF composites. Finally, CQDs, N‐CQDs, S‐CQDs, and NS‐CQDs (10 µg mL^−1^) were assessed using the same protocol to compare their oxidative capacities. To calculate the depletion of GSH, the following equation was employed:

$$\begin{array}{c} \mathrm{loss}\;\mathrm{of}\;\mathrm{GSH}\;\left(\%\right)=\frac{\begin{array}{c} 100\;\times \;\left(\mathrm{absorbance}\;\mathrm{of}\;\mathrm{the}\;\mathrm{negative}\;\mathrm{control}\right.\\ \;-\left.absorbance\;of\;the\;sample\right) \end{array}}{\mathrm{absorbance}\;\mathrm{of}\;\mathrm{the}\;\mathrm{negative}\;\mathrm{control}} \end{array}$$

### 4.9 Electron Paramagnetic Resonance (EPR)

Electron paramagnetic resonance (EPR) spin‐trapping technique was used to identify the generation of ROS using a Bruker EMXplus‐10/12 spectrometer (Bruker, Germany). Following UVA treatment of the NS‐CQDs‐RF composites (containing 100 µg mL^−1^ RF) or RF (100 µg mL^−1^) for various time intervals (0, 10, 20, and 30 min), ·O_2_^−^ and ·OH were detected using DMPO as the spin‐trapping reagent, and ^1^O_2_ was detected using TEMP as the spin‐trapping reagent.

### 4.10 Confocal Microscopy

After treatment with different absorption protocols, the central cornea (6 mm in diameter) was promptly excised from the rabbit eye and vertically immersed in OCT embedding medium. The samples were then cut into 10 µm slices via a cryostat (CryoStar NX50, Thermo Scientific). Excitation of RF fluorescence occurred at 488 nm, with emissions recorded from 500 to 560 nm. The acquisition parameters for the three groups in an experiment were uniform. The intensity of RF was quantified by averaging the signal using the Fiji ImageJ software.

In addition, to confirm that NS‐CQDs and NS‐CQDs‐RF could penetrate the corneal epithelium and reach the corneal stroma in the NS‐CQDs (ion) and NS‐CQDs‐RF (ion) groups, NS‐CQDs fluorescence was excited at 405 nm, and emissions were obtained in the range of 420–500 nm with a laser scanning confocal microscope (Zeiss LSM 880 NLO with AiryScan, Germany).

### 4.11 Cobalt Blue Slit Lamp Photography

To evaluate corneal RF penetration across various treatment groups, cobalt blue slit lamp photography was employed. In brief, immediately after treatment, green fluorescence was documented via a slit‐lamp biomicroscope (SLM‐7E, Kanghua, China) under cobalt blue light, employing a narrow beam at a 10× magnification.

### 4.12 Quantitative Analysis of RF Concentration in Corneal Homogenates

To quantify RF content within the corneal stroma in different groups, central corneas (9 mm in diameter, epithelium removed) were immediately harvested from rabbit eyes following different penetration protocols. The tissues were ground in 500 µL of PBS at 60 Hz for 10 min using a cryogenic tissue grinder (JXFSTPRP‐CL, Jingxin, Shanghai, China). The homogenates were then centrifuged at 12 000 rpm for 10 min at 4°C, and the supernatants were collected and promptly tested using a microplate reader (Spectramax M5, Molecular Devices, USA).

### 4.13 In Vivo CXL Evaluation

Male New Zealand White rabbits (2.5–3.0 kg, 4 months old) were housed with food and water provided ad libitum throughout the study period. Initially, complete ophthalmic examinations with slit‐lamp biomicroscopy (SLM‐7E, Kanghua, China) were performed on all rabbits to ensure they were free of ocular disease. Subsequently, the rabbits were anesthetized by intramuscular injection with xylazine hydrochloride (20 mg kg^−1^) and pentobarbital sodium (30 mg kg^−1^).

Male New Zealand White rabbits were stochastically grouped as follows: NS‐CQDs‐RF (ion) group (epi‐on, presoaked with NS‐CQDs‐RF containing 1 mg mL^−1^ RF in ddH_2_O via iontophoresis for 10 min), RF (ion) group (epi‐on, presoaked with 1 mg mL^−1^ RF in ddH_2_O via iontophoresis for 10 min), RF (epi‐off) group (epi‐off, presoaked with 1 mg mL^−1^ RF in 20% Dextran T500 solution for 30 min), Blank group (no treatment). After being presoaked, the corneas were irradiated for 30 min using a solid‐state UVA illuminator, covering an area of 9.0 mm in diameter with an energy delivery of 3 mW cm^−2^.

To investigate the effect of reducing the total UVA energy on the corneal cross‐linking effect when using NS‐CQDs‐RF as photosensitizers, various UVA irradiation protocols were designed for the NS‐CQDs‐RF (ion) group. Specifically, the corneas were exposed to UVA light at 3 mW cm^−2^ for 10, 15, and 30 min.

### 4.14 Stress–Strain Measurement

After different treatments, corneal discs accompanied by a 1 mm‐wide scleral rim were harvested. The samples were then cut into 1 mm‐wide corneal strips using a custom‐designed double‐edged razor blade. Each strip was vertically affixed in the pneumatic jaws of a tensile testing apparatus (Instron 68TM‐30, Instron INC., MA, USA). After preconditioning the strips through three cycles of stretching under a force of 0.5 N at a 2 mm min^−1^ rate, the strips were subsequently stretched at the same loading rate until a 20% deformation was achieved. Then, stress–strain curves were generated, and the Young′s modulus was determined by analyzing the slope of the linear portion of each curve, with results expressed in MPa [54].

### 4.15 Enzymatic Digestion of Collagen

After various treatments, the central cornea (9 mm in diameter) was excised using a trephine and immersed in 2 mL of 0.1% Collagenase Type II solution. The sample was kept under constant shaking at 200 rpm and 37°C until complete digestion occurred. During digestion, the remaining corneal tissues were monitored at specific time intervals by capturing images. The corneal disc area was assessed using Fiji Image J software, and a curve of the residual corneal disc area was plotted as a function of time. Meanwhile, the time of complete digestion of corneal discs was recorded for different groups.

### 4.16 Anterior Segment Optical Coherence Tomography

To assess the extent of CXL, the stromal demarcation line was detected in enhanced horizontal meridian images of the cornea using anterior segment optical coherence tomography (AS‐OCT) (RTVue XR, Optovue Inc., Fremont, CA, USA). When the corneal reflex was visible, the image was photographed, and the depth of the demarcation line was determined with the caliper tool included in the software. Analysis was conducted on the central cornea, extending from the epithelial side to the hyperreflective line in the stroma, in all groups at 3 weeks postoperatively. Additionally, the demarcation line depth (1 mm to the right and left of the center) was also evaluated. The depth of the demarcation line was assessed by two independent observers.

### 4.17 DFT Calculations Detail

DFT calculations were performed using VASP with the PBE‐GGA functional [65]. Convergence thresholds for geometric optimization were set to 10^−5^ eV for energy and 0.03 eV Å^−1^ for force. Bader charge analysis was used to quantify interfacial electron redistribution [66]. The HOMO/LUMO energy levels and TDOS/PDOS were computed to elucidate the interfacial energy alignment and electronic structures [67]. Free energies for O_2_ adsorption and ^1^O_2_ desorption were evaluated using the optimized models [68]. Finally, TD‐DFT was employed to determine the S1/T1 excited‐state energy levels and the singlet‐triplet gap (E_S1−T1_) [67], assessing the thermodynamic feasibility of ^1^O_2_ generation.

### 4.18 Statistical Analysis

All experiments included a minimum sample size of *n* ≥ 3. Statistical analyses were performed using GraphPad Prism 8.0 (GraphPad Software, La Jolla, CA, USA). Data were presented as means ± standard deviation (means ± SD). Differences between two groups were analyzed using two‐tailed unpaired Student's *t*‐tests, while multiple groups were assessed via one‐way analysis of variance (ANOVA) with Tukey's post hoc test. Additionally, a two‐way ANOVA was utilized to assess the impact of two independent variables on a continuous variable. Statistical significance was set at *p* < 0.05, with varying levels indicated as follows: ^*^ *p* < 0.05, ^**^ *p* < 0.01, and ^***^ *p* < 0.001.

## Author Contributions

T.X. and Y.L. contributed equally to this work. Y.L. and S.C. conceived the idea. T.X., Y.J., and M.L. designed and carried out the experiments, performed data analysis, and visualized the data. T.X., W.Z., and Q.J. synthesized the materials and conducted material characterization. L.L., L.W., and Z.G. provided important experimental insights. Y.L. and S.C. supervised the project. T.X. drafted the manuscript. Y.L. and S.C. performed critical review and editing. All the authors discussed, commented, and agreed on the manuscript.

## Conflicts of Interest

The authors declare no conflicts of interest.

## Supporting information




**Supporting File**: advs75561‐sup‐0001‐SuppMat.docx.

## Data Availability

The data that support the findings of this study are available from the corresponding author upon reasonable request.
