# Supplementary material for: N, S Co‐Doped Carbon Quantum Dots‐Riboflavin Composite Photosensitizers for Enhanced Iontophoresis‐Assisted Corneal Cross‐Linking
Source: Adv Sci (Weinh). 2026 May 5;13(42):e10396. doi: 10.1002/advs.202510396 (PMC13335933; doi:10.1002/advs.202510396)
Supplement: Supplementary file 1 — Supporting File: advs75561‐sup‐0001‐SuppMat.docx. [file ADVS-13-e10396-s001.docx]

Supporting Information

N, S Co-Doped Carbon Quantum Dots-Riboflavin Composite Photosensitizers for Enhanced Iontophoresis-Assisted Corneal Cross-Linking

Tinghong Xu^1,2,3,†^, Yong Liu^1,2,†,^*, Wenjing Zhang^1,2^, Qiuruo Jiang^1,3^, Yi Jin^1,3^, Liangzheng Lin^1,3^, Lumeng Wang^1,3^, Zhanhao Gu^1,3^, Mimi Lin^1,2,^*, and Shihao Chen^1,3,^*

T. Xu, Y. Liu, W. Zhang, Q. Jiang, Y. Jin, L. Lin, L. Wang, Z. Gu, M. Lin, S. Chen

^1^School of Ophthalmology and Optometry, School of Biomedical Engineering, Wenzhou Medical University, Wenzhou, Zhejiang 325027, China

*E-mail: yongliu@wmu.edu.cn; linmimi815@wmu.edu.cn; csh@eye.ac.cn

T. Xu, Y. Liu, W. Zhang, M. Lin

^2^Laboratory of Novel Optoelectronic Technology for Ophthalmic Devices (NOTOD), National Engineering Research Center of Ophthalmology and Optometry, Eye Hospital, Wenzhou Medical University, Wenzhou, Zhejiang 325027, China

T. Xu, Q. Jiang, Y. Jin, L. Lin, L. Wang, Z. Gu, S. Chen

^3^The Institute of Ocular Biomechanics, National Clinical Research Center for Ocular Diseases, Eye Hospital, Wenzhou Medical University, Wenzhou, Zhejiang 325027, China

†These authors contributed equally to this work.

**Supplementary Materials and Methods**

**Study Design**

The objective of this study was to develop N, S co-doped carbon quantum dots-riboflavin composite photosensitizers (NS-CQDs-RF) for enhanced iontophoresis corneal cross-linking (I-CXL). First, we prepared NS-CQDs-RF composite photosensitizers and conducted material characterization and property studies. Then, we evaluated the biocompatibility and riboflavin permeation capabilities of NS-CQDs-RF at the cellular level. After that, we investigated the corneal riboflavin penetration, reactive oxygen species generation capacity, and biosafety of NS-CQDs-RF in rabbit eyes. Finally, we comprehensively assessed the therapeutic efficacy of NS-CQDs-RF applied in I-CXL in rabbit eyes.

**In Vitro Permeation Experiment**

HCECs were plated into 0.4 μm pore-size Transwell membrane inserts (Coster), coated with Collagen Type I (Corning), at a density of 2 × 10^5^ cells per insert and placed in 12-well plates. For cell proliferation, 1.5 mL of DMEM/F12 medium was added to each well, and 0.5 mL of the same medium was added to each insert containing cells. After 5 to 7 days, the cultures underwent an air-lift procedure by removing the medium from the inserts. The cells were then maintained at the air-liquid interface for 1 to 2 weeks until the cultures reached a thickness of 3 to 5 cell layers. The transepithelial resistance (TER) of the multilayer corneal epithelial cell cultures was measured using an EVOM2^TM^ Epithelial Volt/Ohm Meter (World Precision Instruments, USA) [1]. When the TER reached its maximum and stabilized, penetration studies were carried out. For penetration studies, 0.5 mL of RF or NS-CQDs-RF dissolved in DPBS was added to the inserts, and 1 mL of DPBS was added to the wells. Subsequently, electrical stimulation at a current of 1 mA was applied to the inserts. At specific time intervals, samples from the wells were collected and analyzed by a microplate reader (Spectramax M5, Molecular Devices, USA).

**Cytocompatibility of NS-CQDs-RF Composites**

Human corneal epithelial cells (HCECs) were provided by the American Type Culture Collection (ATCC, USA). Rabbit corneal stromal cells (RCSCs) were obtained from Saibaikang Biotechnology Co. Ltd (Shanghai, China).

*Live/Dead Cell Staining*

First, HCECs and RCSCs were placed in 96-well plates with a density of 1 × 10^4^ cells per well and cultured for 24 h. Then, NS-CQDs-RF (1 mg mL^−1^ RF) were introduced to the wells for another 24 h of incubation. Finally, staining of the cells was performed with Calcein-AM/PI, and representative images were captured by an inverted fluorescence microscope (Zeiss Axio Observer, Germany).

*Cell Counting Kit-8 (CCK-8)*

In summary, HCECs and RCSCs were seeded at 1 × 10^4^ cells per well in 96-well plates and grown overnight. Subsequently, different concentrations of NS-CQDs-RF were applied, and the cells were incubated for an additional 24 h. Afterwards, 10 μL of CCK-8 solution was added to each well, and was incubated at 37°C for 1 to 2 h shielded from light. The absorbance at 450 nm was detected using a microplate reader (Spectramax M5, Molecular Devices, USA).

*Annexin V Apoptosis Assay*

Cell apoptosis after coincubation with the materials was investigated using the Annexin V-FITC/PI kit (BD Biosciences, USA). HCECs and RCSCs were seeded in six-well plates at a density of 4 × 10^5^ cells per well and grown overnight, and then incubated with NS-CQDs-RF (1 mg mL^−1^ RF) for 24 h. After trypsin digestion, the cells were washed with DPBS and resuspended in 400 μL of 1× binding buffer. Subsequently, the cells were sequentially stained with 5 μL of Annexin V-FITC and 5 μL of PI. After a 15-minute incubation (protected from light), samples were assessed using a BD Accuri C6 Plus flow cytometer (BD Biosciences, USA).

**Evaluation of ROS Production in Corneal Homogenates**

Immediately after absorption with various treatments, the central corneas (9 mm in diameter, epithelium removed) were harvested for homogenization as previously mentioned. The supernatant from the homogenates was then exposed to UVA light at 3 mw cm^−2^ for 30 min in the presence of Rb, DPBF, and GSH, which function as ROS trapping agents, as detailed in the previously established protocol.

**In vivo CXL evaluation**

*Transmission Electron Microscopy*

Male New Zealand White rabbits were euthanized one month after corneal cross-linking. Small tissue blocks were excised from the central portion of the corneal strips and immediately fixed in fresh TEM fixation. Then, the fixed samples underwent a series of treatments, including agarose pre-embedding, post-fixation, dehydration, resin infiltration, embedding in LX-112 resin, polymerization, ultrathin sectioning, and staining. The copper grids were examined by a transmission electron microscope (Hitachi, Japan, H7500 TEM), and images were acquired. Ultrastructural parameters evaluated included collagen fibril diameter, interfibrillar spacing, and periphery-to-periphery spacing.

**In vivo biocompatibility**

*Corneal Thickness and Intraocular Pressure Measurements*

Central corneal thickness and limbal thickness were measured with an ultrasound pachymeter (PachPen; Accutome, Malvern, PA, USA) before treatment and weekly until the treatment was ended at 4 weeks. At the same time points, intraocular pressure was measured using a tonometer (Tono-Pen AVIA Vet, USA).

*Hematoxylin-Eosin Staining*

Following the respective treatments, the corneas and major organs (heart, liver, spleen, lung, and kidney) of the rabbits were harvested and fixed in 4% PFA for 24 h. The specimens were then embedded in paraffin, sectioned at a thickness of 5 μm, and mounted onto glass slides. After standard dewaxing and rehydration, hematoxylin-eosin (H&E) staining was performed to evaluate the corneal morphological changes and systemic biosafety across the four groups. Finally, the stained sections were imaged using a Pannoramic Scanner (3D Pannoramic, Belgium).

*Masson’s Trichrome Staining of Cornea*

To observe the corneal stroma, sections were stained using Masson’s trichrome stain kit following the manufacturer’s instructions (Solarbio, China). After deparaffinization, the sections were sequentially stained with an array of staining solutions, including Weigert’s iron hematoxylin, acid differentiation, bleaching, ponceau-acid fuchsin, phosphoric acid, and aniline blue solutions. Subsequently, the images were visualized with a Pannoramic Scanner (3D Pannoramic, Belgium).

*TUNEL Staining of Cornea*

The TUNEL assay was performed following the manufacturer’s instructions, using the one-step TUNEL Assay Apoptosis Detection Kit (Beyotime, China). In brief, central corneas were harvested at 2, 4 and 12 weeks after treatment, embedded in optimal cutting temperature (OCT) compound, and sectioned into 10-μm slices. The sections were then fixed with 4% PFA at room temperature for 15 min. After washing twice with PBS, the samples were permeabilized with 0.5% Triton X-100 for 5 min. Subsequently, 50 μL of the TUNEL reaction mixture (containing 45 μL labeling solution and 5 μL TdT enzyme solution) was applied, and the sections were incubated at 37°C for 1 h in a dark, humidified chamber. The sections were then counterstained with DAPI and visualized using a laser scanning confocal microscope (Zeiss LSM 880 NLO with AiryScan, Germany). The TUNEL-positive control group was prepared by incubating the sections with DNase I at room temperature, utilizing the TUNEL-positive control kit (Beyotime, China).

*Slit Lamp Biomicroscopy*

At 2, 4 and 12 weeks post-treatment, ocular manifestations were assessed using a slit lamp (SLM-7E, Kanghua, China), and epithelial integrity was assessed using fluorescein staining. A volume of 5 μL of sodium fluorescein solution was administered into the conjunctival sacs of the rabbits, followed by 2–3 manual blinks. After a 2-minute interval, corneal fluorescein staining was examined under cobalt blue light.

*Endothelium Staining*

After 2, 4 and 12 weeks of CXL, the central 6 mm of the cornea was trephined, taking care to avoid mechanical damage to the corneal endothelium. The corneas were then placed on adhesive microscope slides with the endothelium facing up. Then, the endothelium was covered with 0.2% trypan blue for 100 s and washed twice with PBS. After that, the endothelium was covered with Alizarin red S solution (1%, pH 4.2) for another 100 s and rinsed twice with PBS. After the sections were thoroughly air-dried, endothelial morphology was visualized using a microscope (Leica DM4 B), and corneal endothelial cell density was recorded.

**Supplementary Figures**

**Figure S1.** Encapsulation and loading efficiencies of RF in NS-CQDs-RF (*n* = 3). Data are means ± SD.

**Figure S2.** Zeta potential of NS-CQDs-RF in double-distilled H_2_O (ddH_2_O) with time-dependent variations (*n* = 4). Data are means ± SD.

**Figure S3.** UV-vis absorption spectra for RF, NS-CQDs, and NS-CQDs-RF.


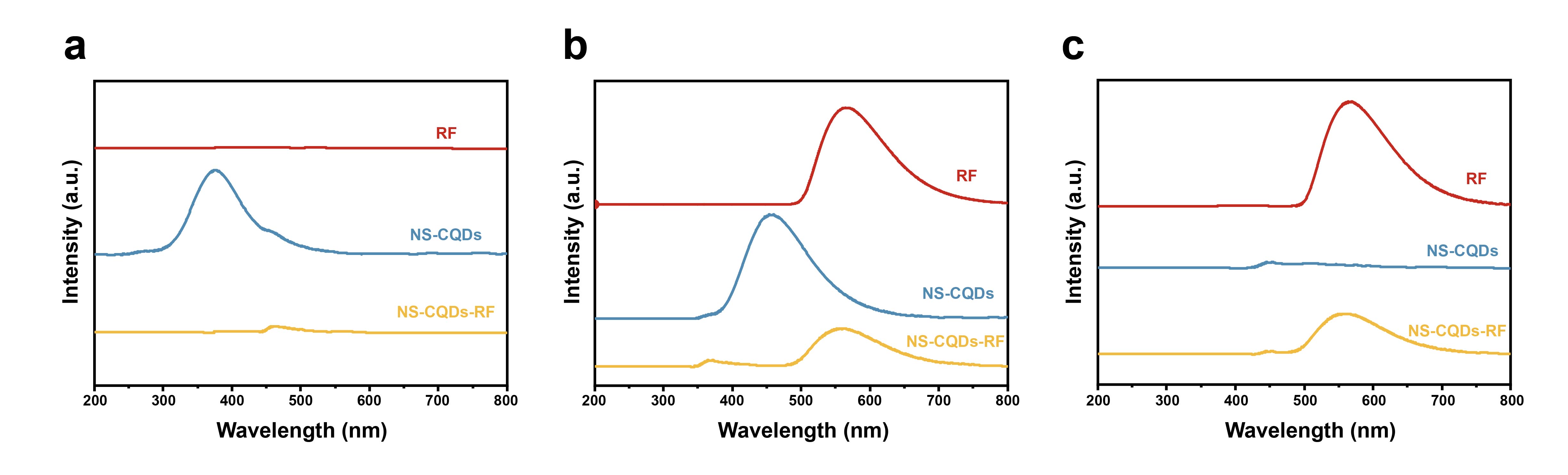


**Figure S4.** Fluorescence spectra for RF, NS-CQDs, and NS-CQDs-RF. (a) Excitation spectra at 450 nm. (b) Emission spectra at 360 nm. (c) Emission spectra at 440 nm.


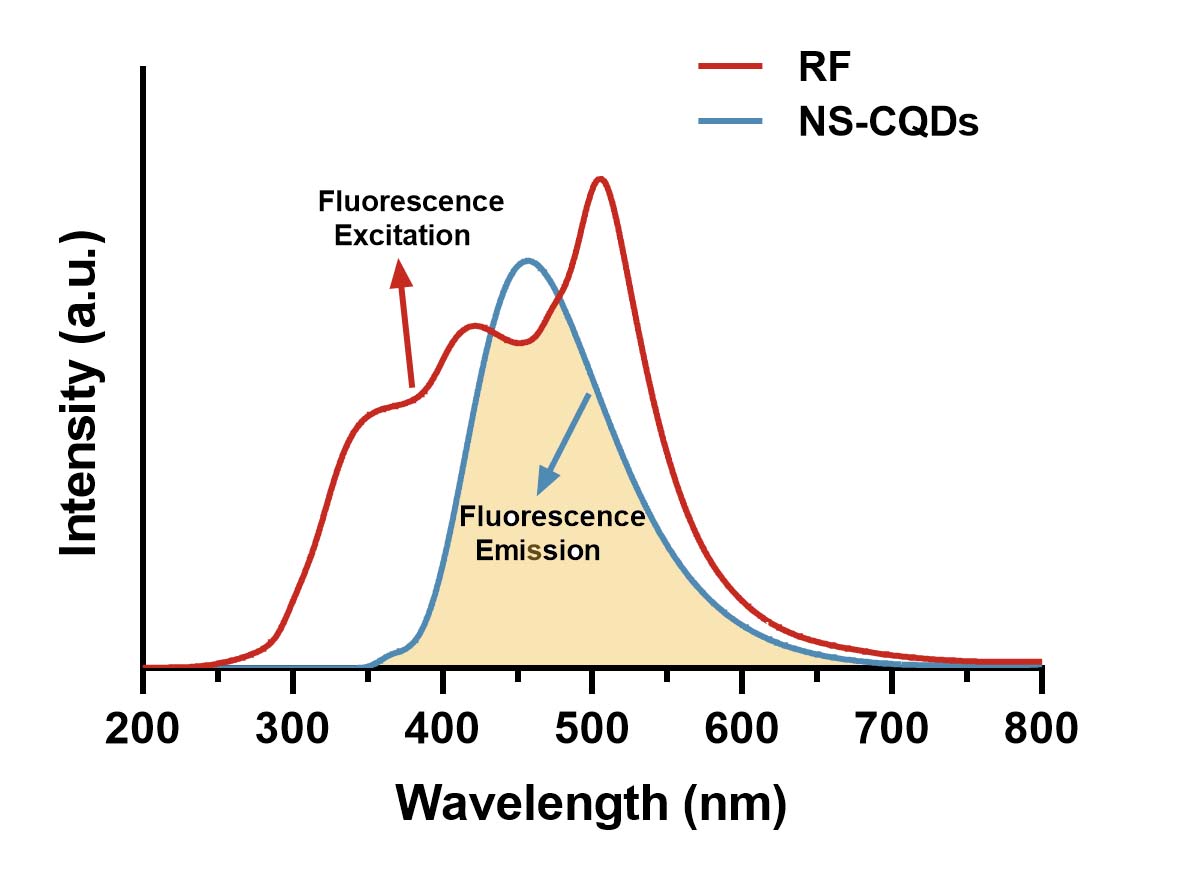


**Figure S5.** Fluorescence excitation spectrum of RF and emission spectrum of NS-CQDs.


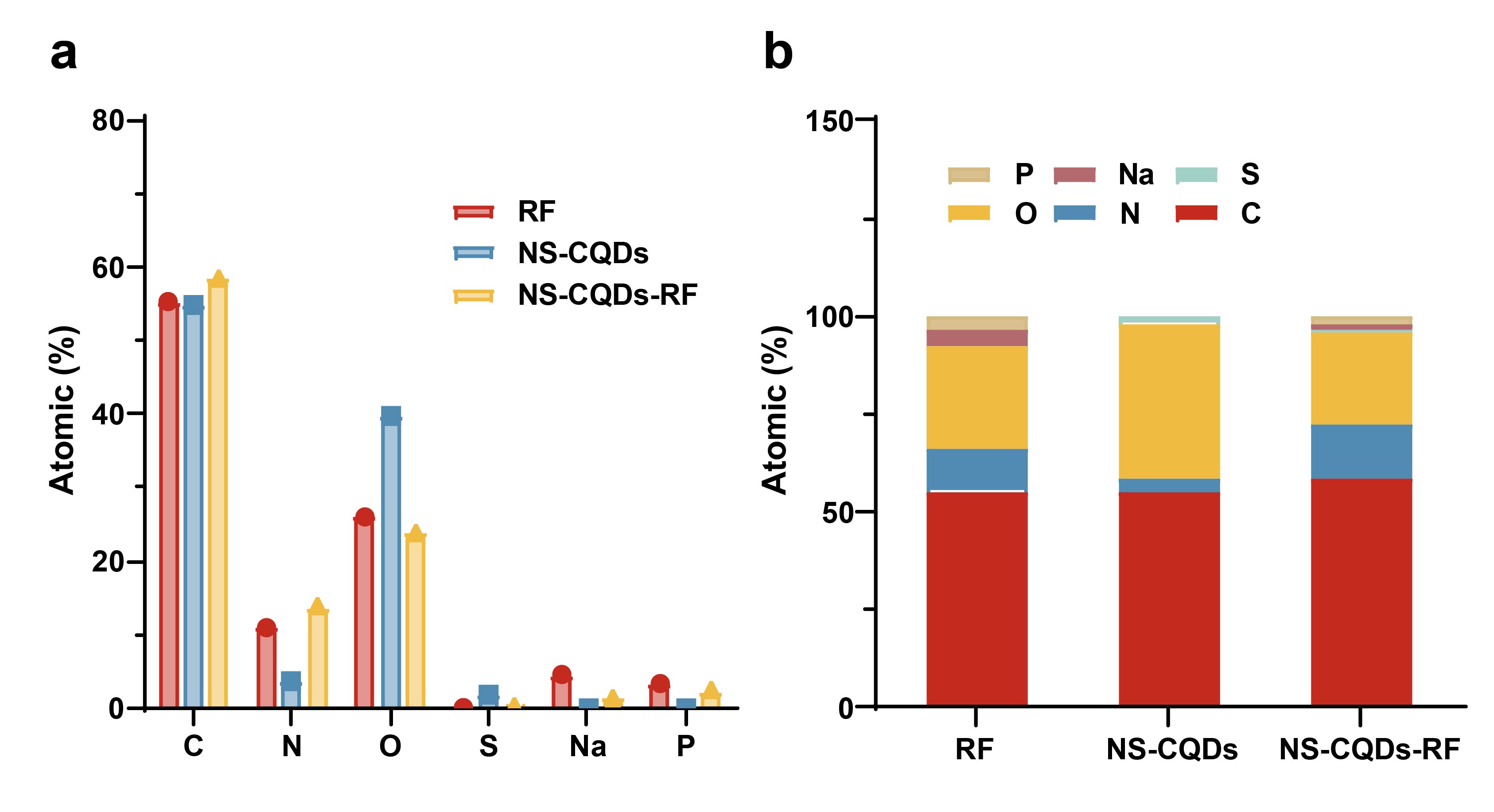


**Figure S6.** Elemental composition analysis of RF, NS-CQDs, and NS-CQDs-RF determined by X-ray photoelectron spectroscopy. (a,b) Graphs of the elemental proportions of C, N, O, S, Na, and P in RF, NS-CQDs, and NS-CQDs-RF.

**
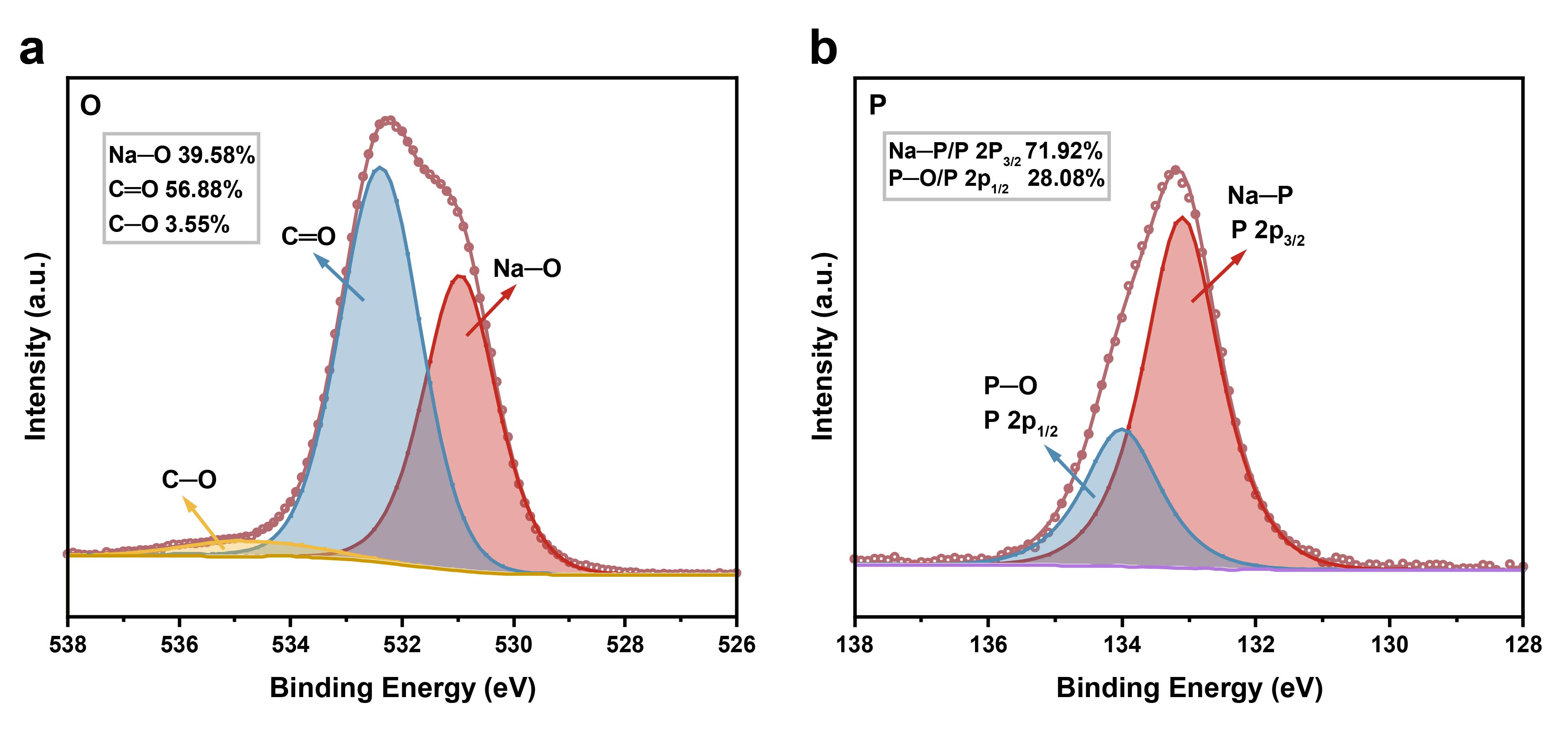
**

**Figure S7.** High-resolution XPS spectra of (a) O 1s and (b) P 2p.

**Figure S8.** UV-vis absorption spectra for CQDs, N-CQDs, S-CQDs, and NS-CQDs.

**
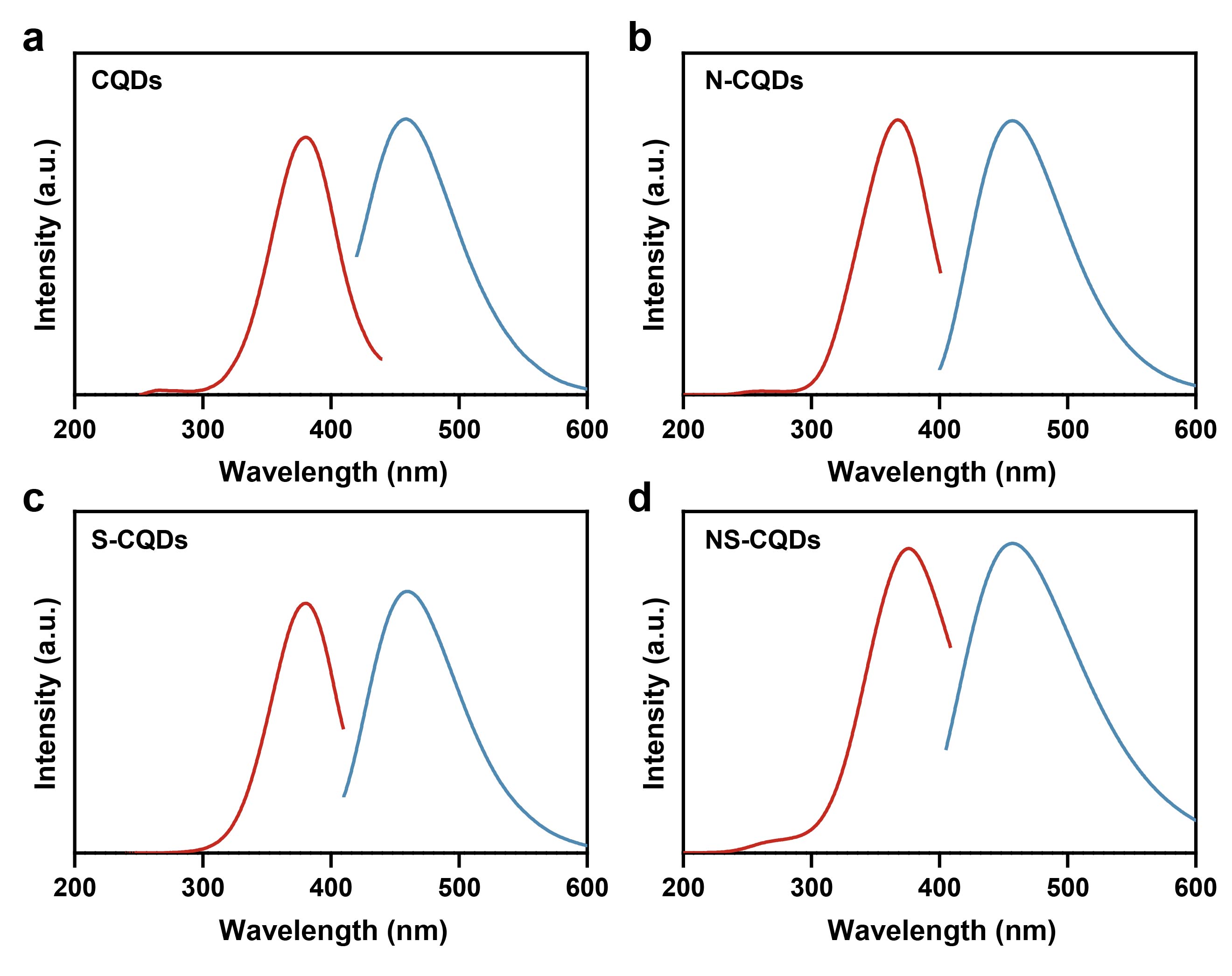
**

**Figure S9.** Excitation (red color) and emission (blue color) spectra of (a) CQDs, (b) N-CQDs, (c) S-CQDs, and (d) NS-CQDs dispersed in double-distilled water (ddH_2_O).

**
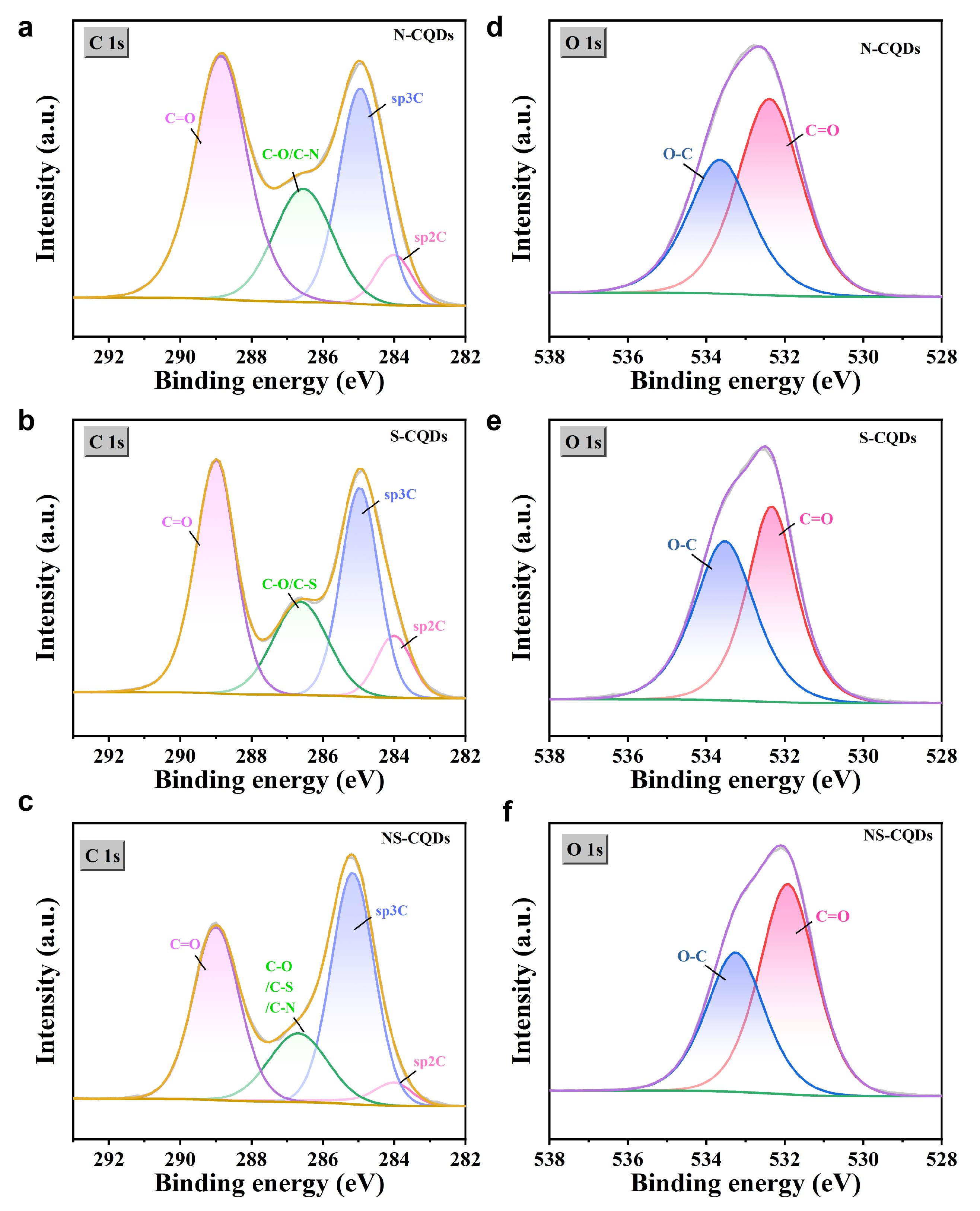
**

**Figure S10.** High-resolution XPS spectra of (a–c) C 1s and (d–f) O 1s for N-CQDs, S-CQDs, and NS-CQDs.

**Figure S11.** High-resolution XPS spectra of (a,b) N 1s and (c,d) S 2p for N-CQDs, S-CQDs, and NS-CQDs.

**
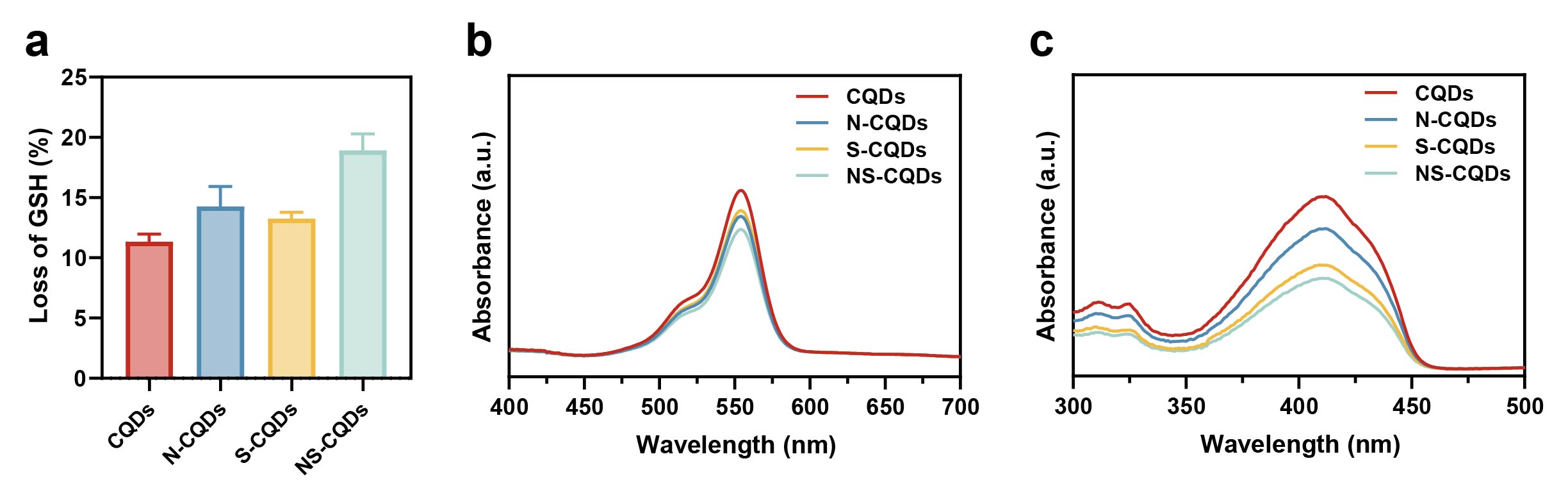
**

**Figure S12.** Comparative evaluation of ROS generation under UVA irradiation. (a) GSH depletion (*n* = 6), and degradation of (b) Rb and (c) DPBF probes by CQDs, N-CQDs, S-CQDs, and NS-CQDs.

**
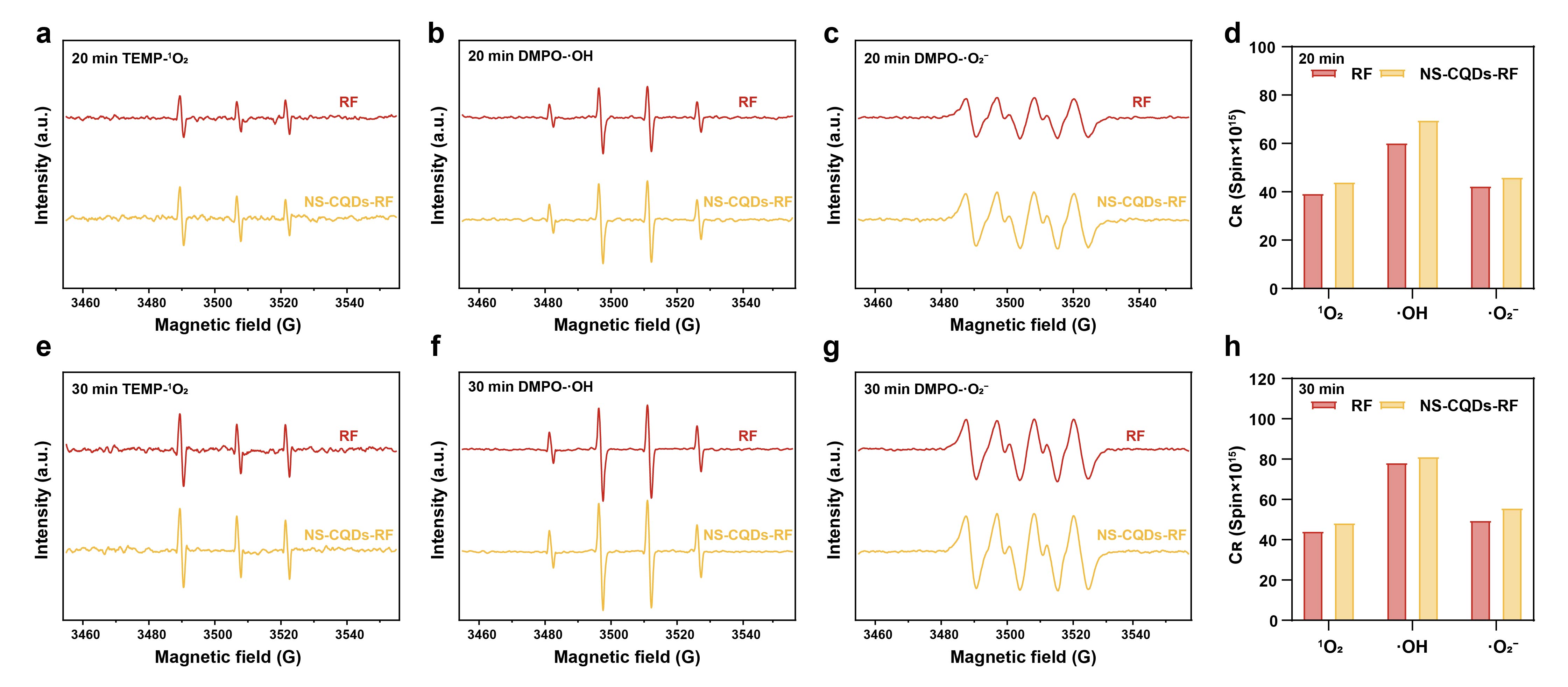
**

**Figure S13.** Electron paramagnetic resonance (EPR) evaluation of ROS generation. (a–c,e–g) EPR spectra of TEMP-^1^O_2_, DMPO-·OH, and DMPO-·O_2_^−^ produced by free RF and NS-CQDs-RF in aqueous solutions after 20 and 30 min of UVA irradiation. (d,h) Concentrations of ^1^O_2_, ·OH, and ·O_2_^−^ (C_R_) at 20 and 30 min, respectively.

**
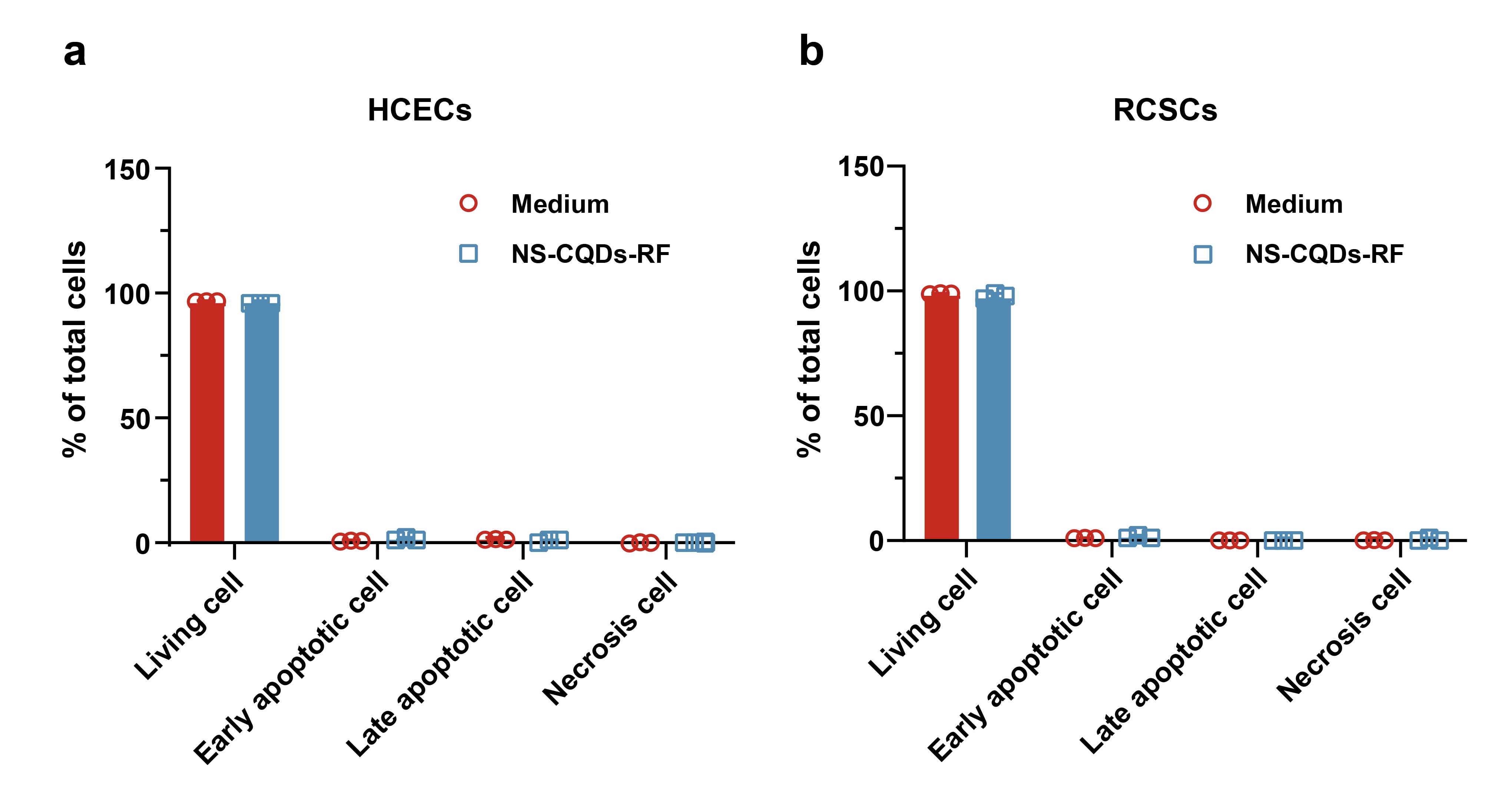
**

**Figure S14.** Statistical analysis of (a) HCECs and (b) RCSCs treated with NS-CQDs-RF for 24 h using Annexin V/PI staining (*n* = 3). Data are means ± SD.

**
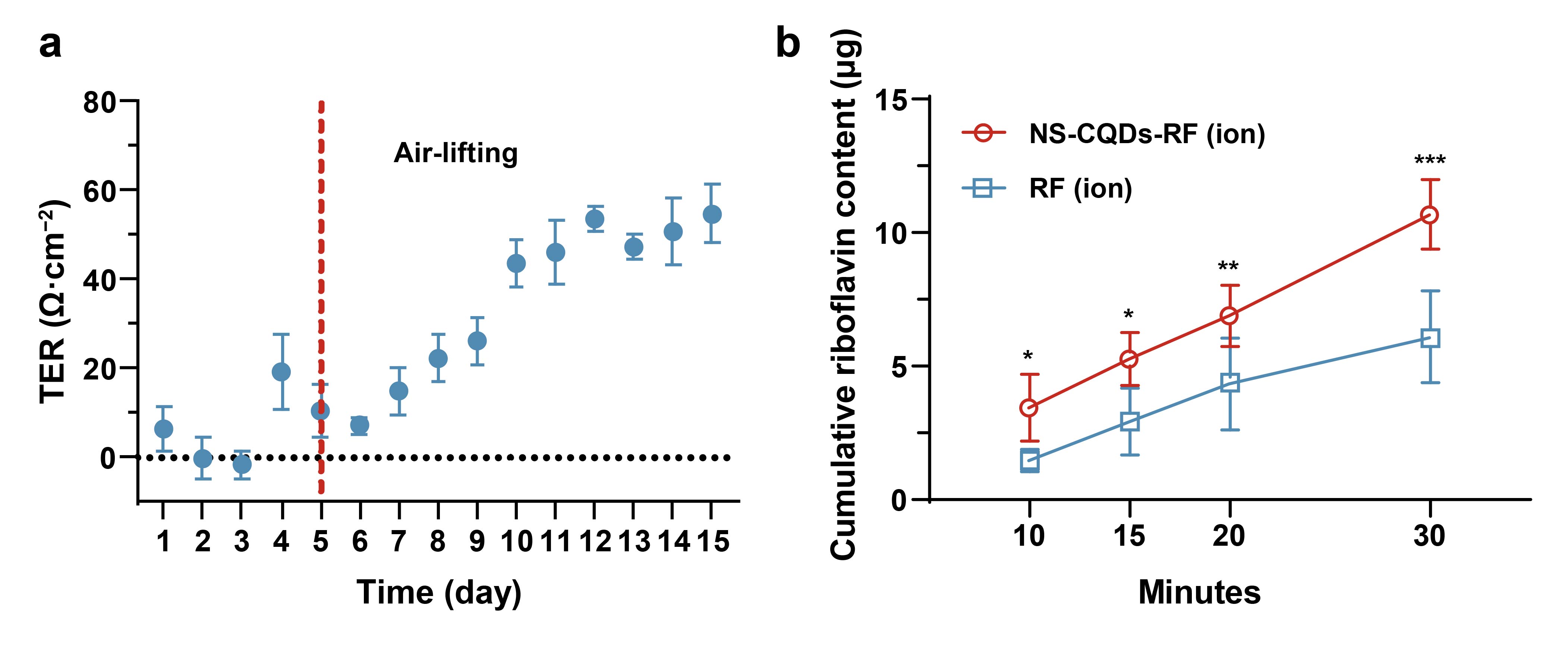
**

**Figure S15.** RF permeability evaluation in a Transwell culture model. (a) Transepithelial resistance (TER) measurements over time (*n* = 12). (b) Quantification of cumulative RF content in the lower chamber as a function of time (*n* = 6). Data are means ± SD. **P* < 0.05, ***P* < 0.01, and ****P* < 0.001. Statistical analysis was performed using two-way ANOVA followed by Bonferroni’s multiple comparisons test. ion, iontophoresis.

**
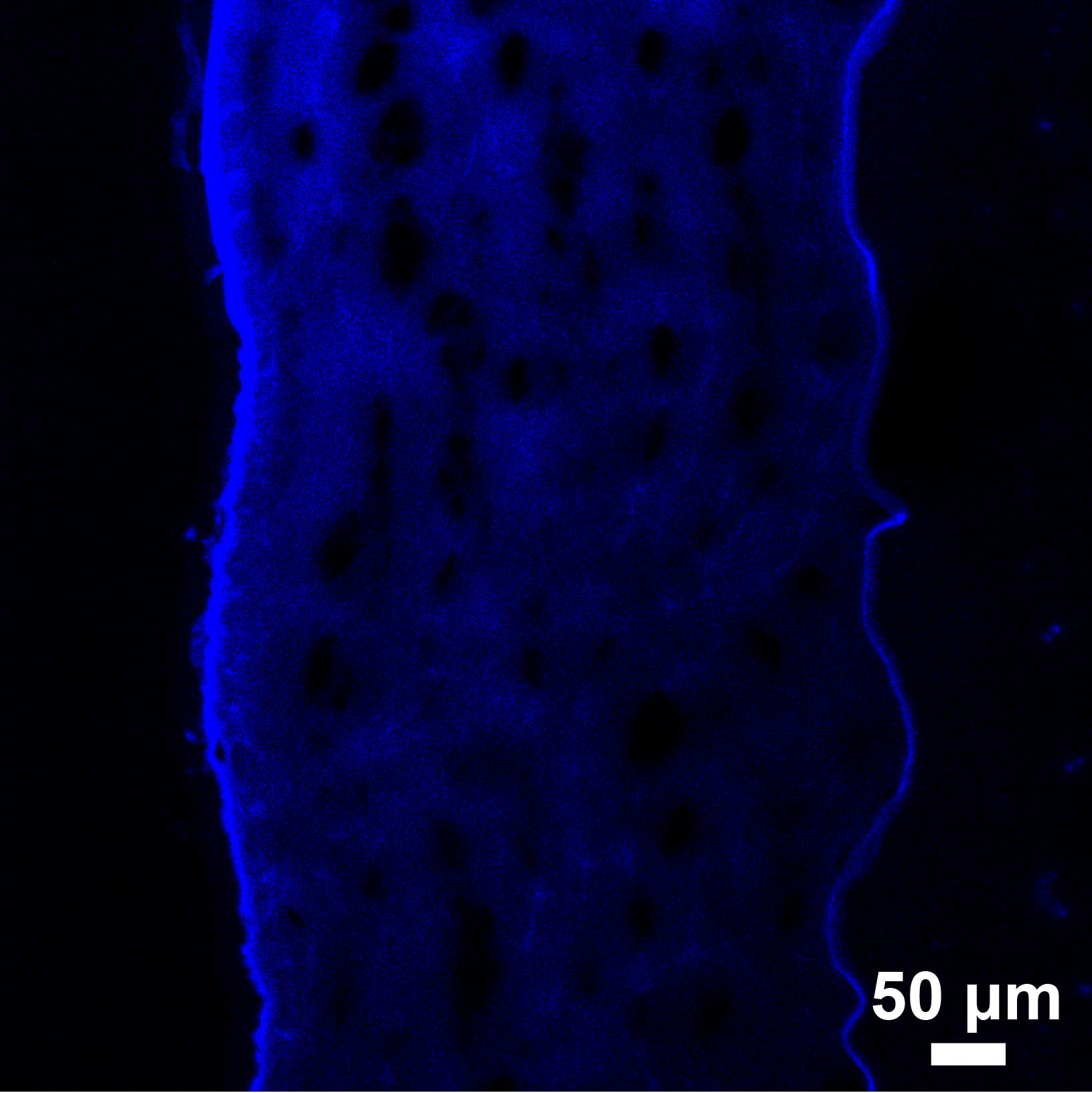
**

**Figure S16.** Confocal fluorescence image of the cornea permeabilized with NS-CQDs by ion introduction. The blue fluorescence indicates the presence of NS-CQDs.

**
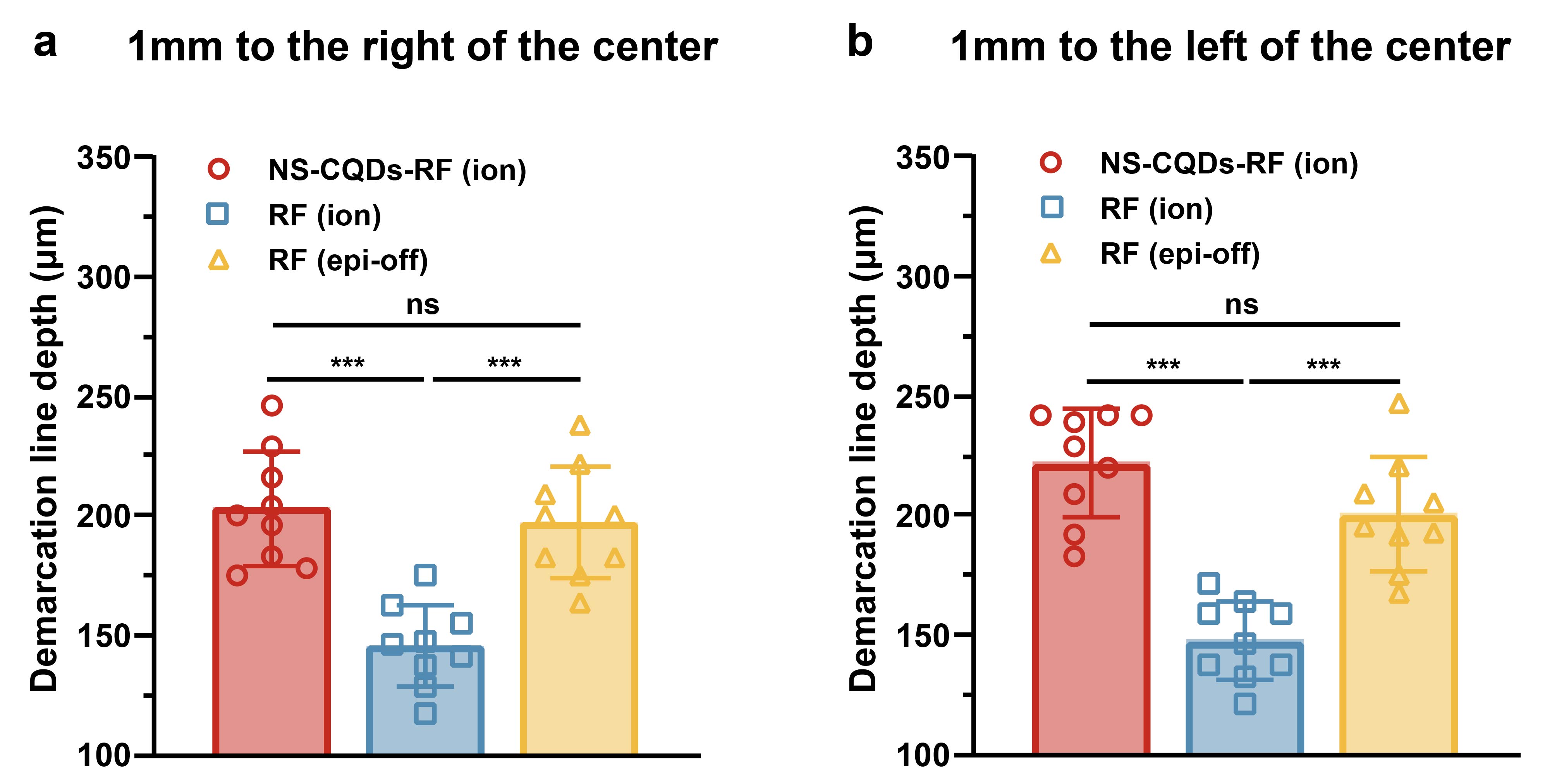
**

**Figure S17.** Statistical analysis of the demarcation line depth. (a) 1 mm to the right of the center (*n* = 9). (b) 1 mm to the left of the center (*n* = 9). Data are means ± SD. ns, no significant difference; ****P* < 0.001. Statistical analysis was performed using one-way ANOVA followed by Tukey’s multiple comparisons test. ion, iontophoresis; epi-off, epithelium-off.

**
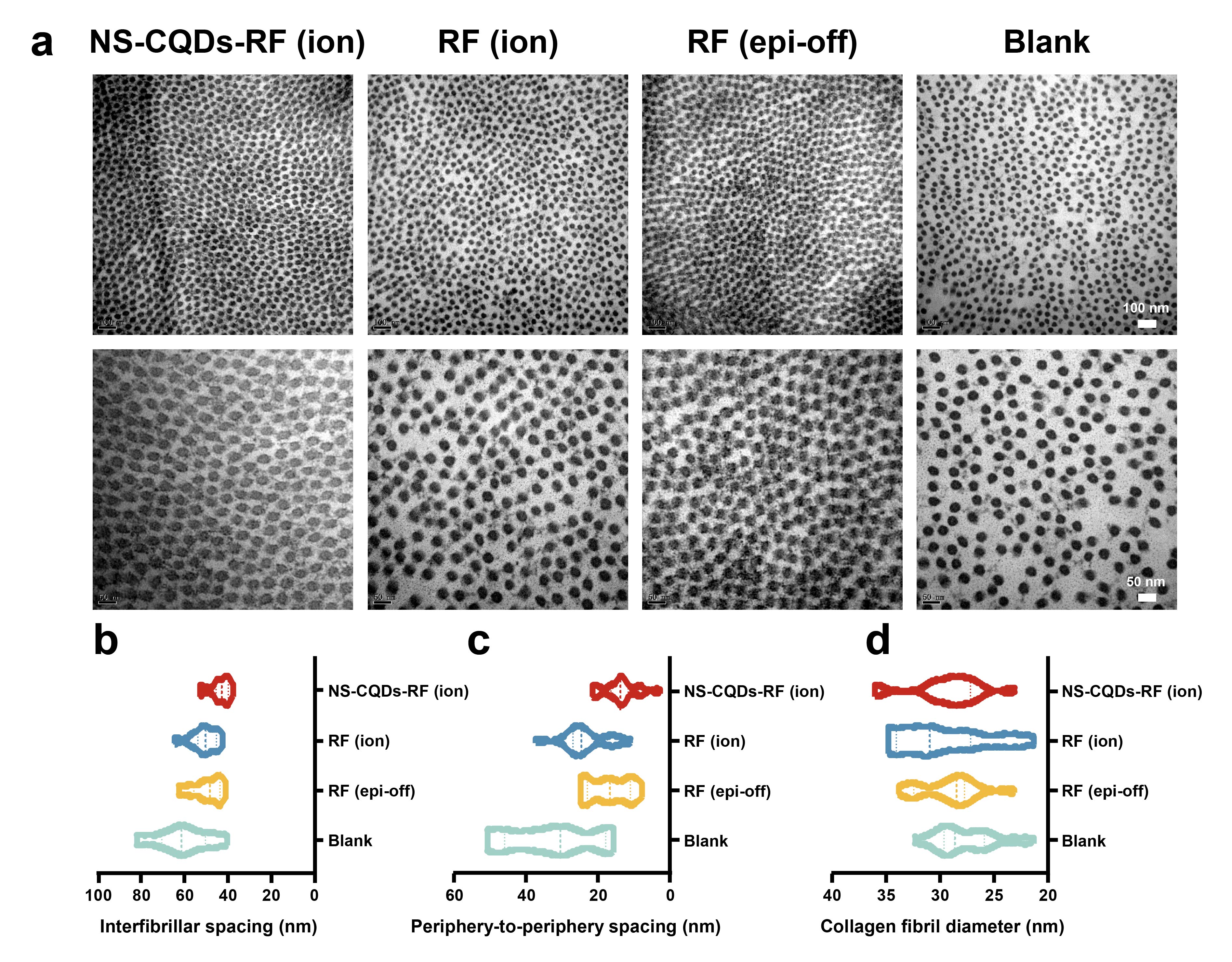
**

**Figure S18.** Transmission electron microscopy (TEM) analysis of the anterior portion of the corneal stroma under different treatments. (a) Representative TEM images. (b) Interfibrillar spacing. (c) Periphery-to-periphery spacing. (d) Collagen fibril diameter. ion, iontophoresis; epi-off, epithelium-off.

**
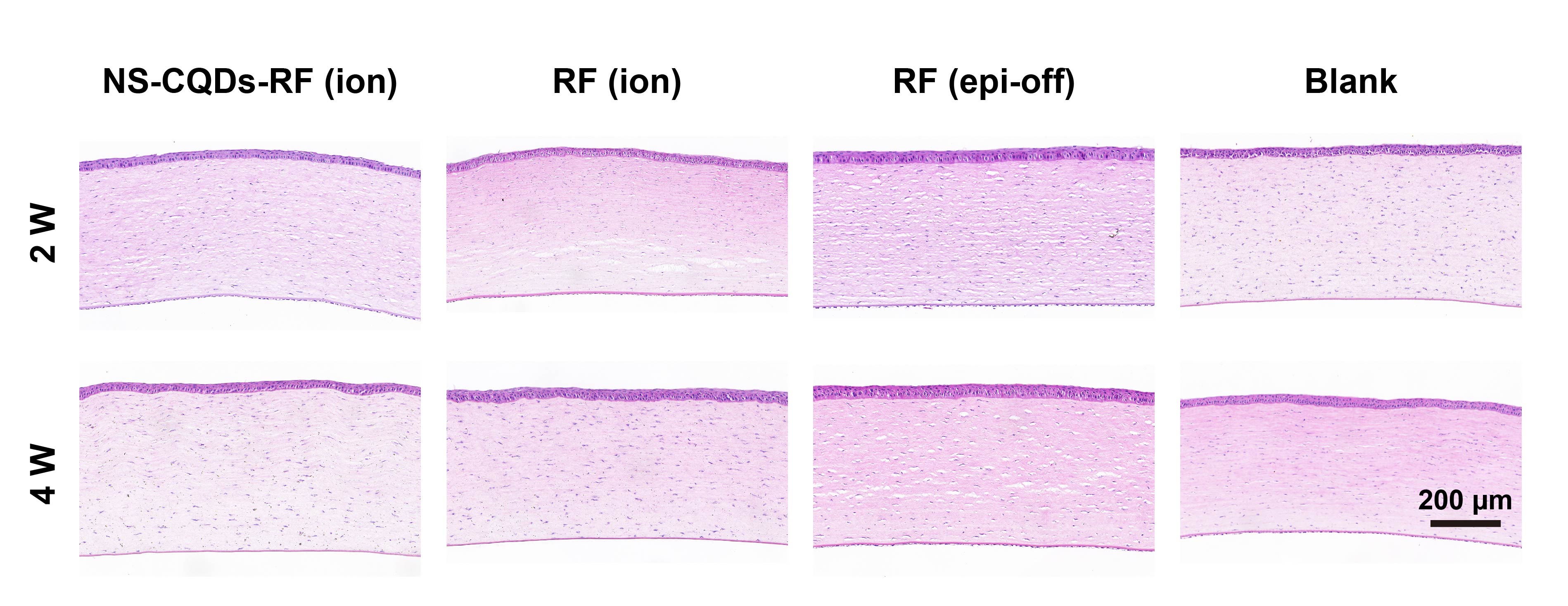
**

**Figure S19.** HE staining at week 2 and 4 after CXL for the NS-CQDs-RF (ion), RF (ion), RF (epi-off), and Blank groups. ion, iontophoresis; epi-off, epithelium-off.

**
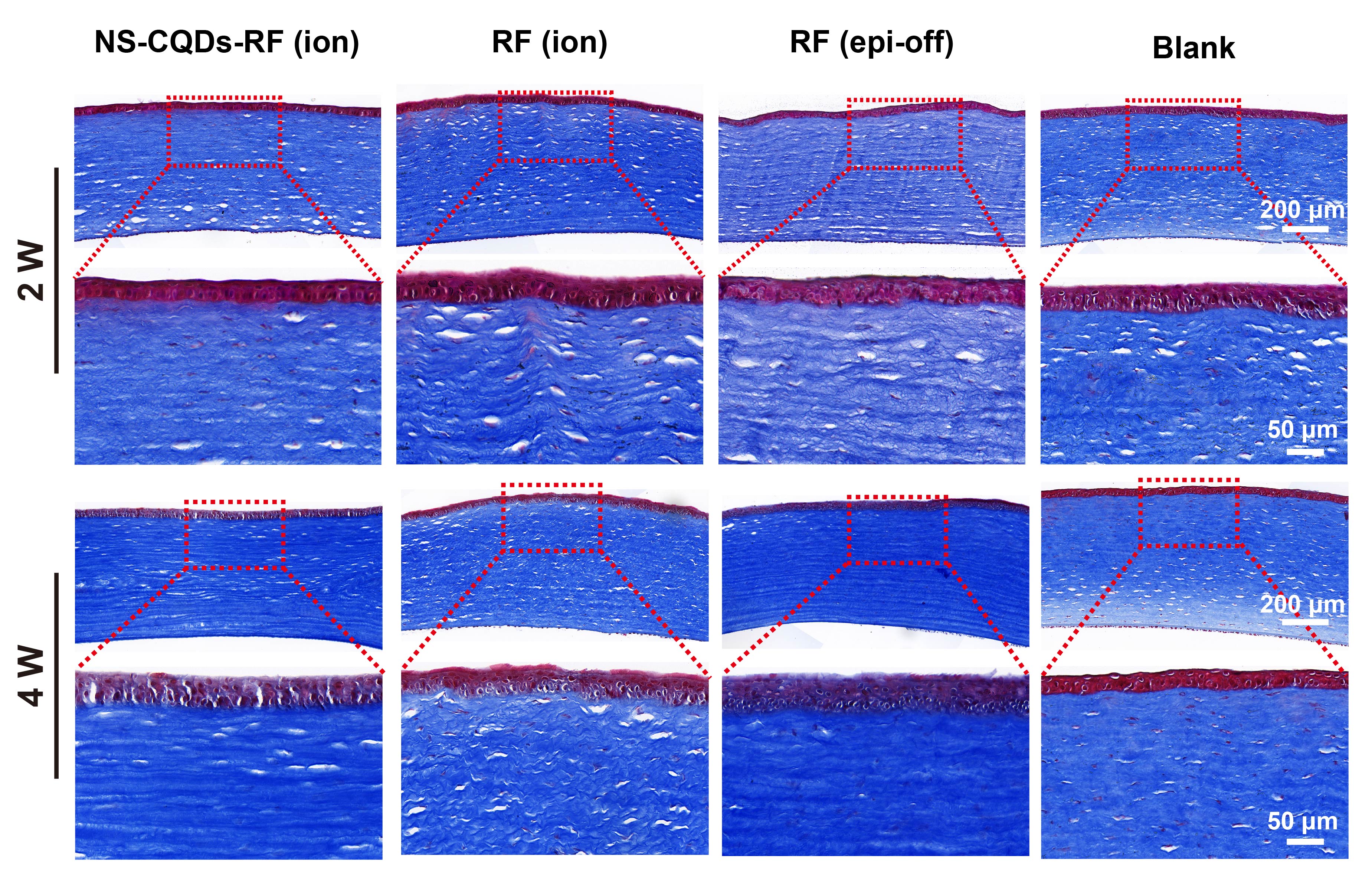
**

**Figure S20.** Masson’s trichrome staining at week 2 and 4 after CXL for the NS-CQDs-RF (ion), RF (ion), RF (epi-off), and Blank groups. ion, iontophoresis; epi-off, epithelium-off.

**
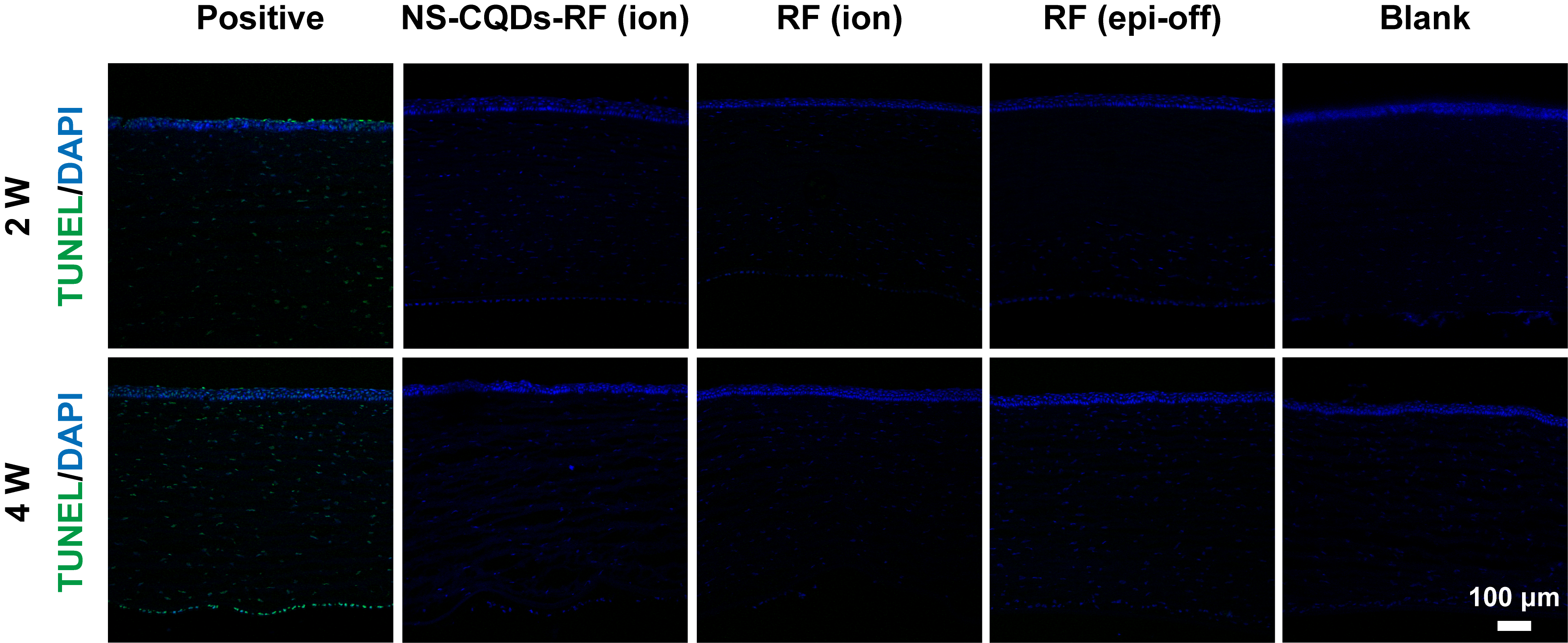
**

**Figure S21.** TUNEL staining at week 2 and 4 after CXL for the Positive, NS-CQDs-RF (ion), RF (ion), RF (epi-off), and Blank groups. Blue fluorescence represents nuclear from DAPI, green fluorescence represents the TUNEL-positive cells. ion, iontophoresis; epi-off, epithelium-off.

**
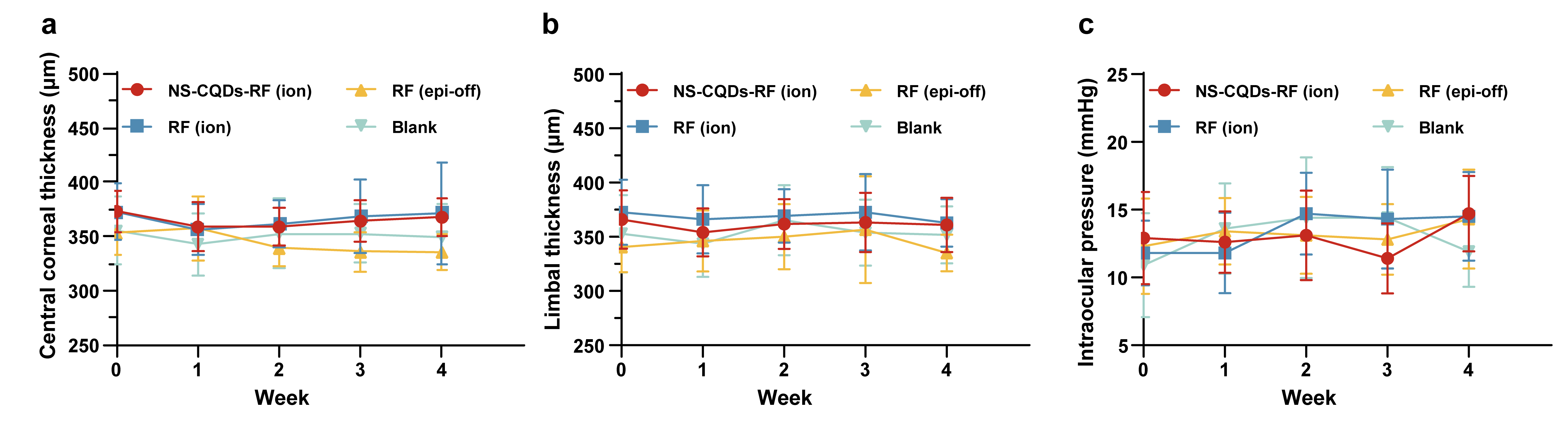
**

**Figure S22.** Postoperative monitoring of ocular physiological parameters. Measurements of (a) central corneal thickness, (b) limbal thickness, and (c) intraocular pressure (*n* = 10). Data are means ± SD. ion, iontophoresis; epi-off, epithelium-off.

**
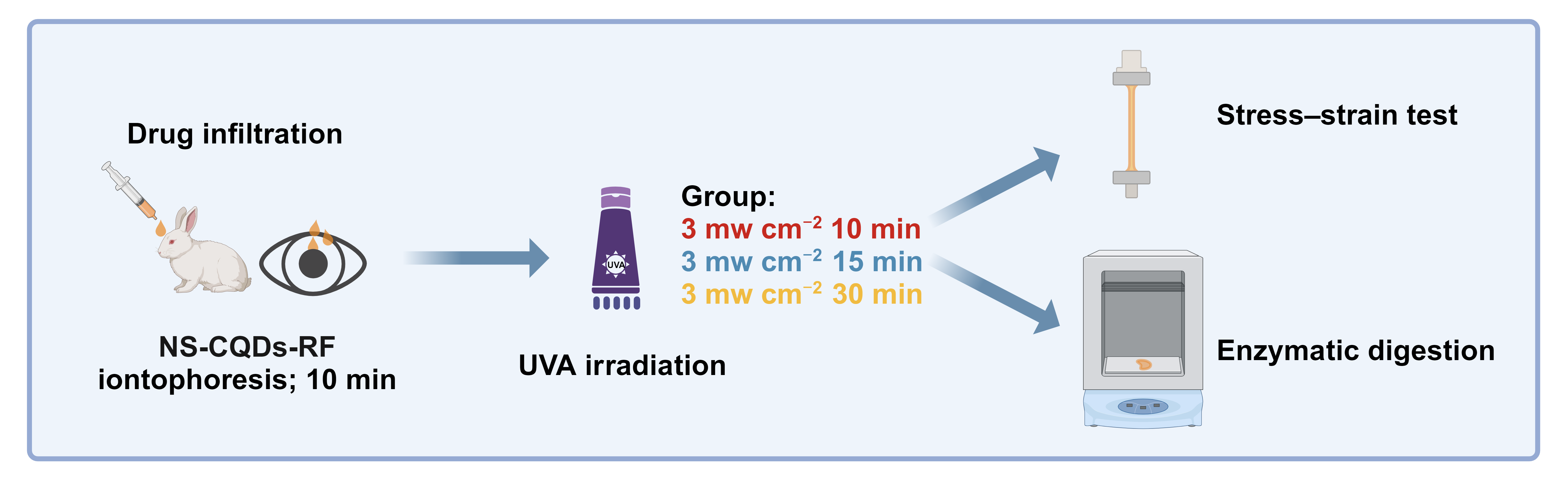
**

**Figure S23.** Flowchart of the efficacy assessment of UVA total energy reduction protocols in I-CXL using NS-CQDs-RF composite photosensitizers. Created with BioRender.com.

**Supplementary Tables**

**Table S1.** XPS Data analyses of the O 1s spectra of three CQDs samples.

| Sample | C─O | C═O |
| --- | --- | --- |
| N-CQDs | 41.22% | 58.78% |
| S-CQDs | 50.15% | 49.85% |
| NS-CQDs | 40.77% | 59.23% |

**References**

1. E. Aytekin, N. Öztürk, İ. Vural, et al., “Design of Ocular Drug Delivery Platforms and In Vitro - In Vivo Evaluation of Riboflavin to the Cornea by Non-Interventional (Epi-On) Technique for Keratoconus Treatment,” *Journal of Controlled Release* 324 (2020): 238–249, https://doi.org/10.1016/j.jconrel.2020.05.017.
